# Morbidity and Complications of Diabetes Mellitus in Children and Adolescents in Ghana: Protocol for a Longitudinal Study

**DOI:** 10.2196/21440

**Published:** 2021-01-06

**Authors:** Vera Adobea Essuman, Naa Naamuah Tagoe, Josephine Akpalu, Akye Essuman, Adziri H Sackey, CF Hayfron-Benjamin, George Asare, Benjamin Abaidoo, AGB Amoah, Thomas Ndanu, IDB Ofori-Adjei, NA Barnes, BL Appiah-Thompson, Winfried M Amoaku

**Affiliations:** 1 Ophthalmology Unit Department of Surgery University of Ghana Medical School Accra Ghana; 2 Eye Department Korle Bu Teaching Hospital Accra Ghana; 3 Department of Medicine and Tharapeutics University of Ghana Medical School Accra Ghana; 4 Department of Community Health University of Ghana Medical School Accra Ghana; 5 Department of Child Health University of Ghana Medical School Accra Ghana; 6 Department of Physiology University of Ghana Medical School Accra Ghana; 7 Department of Anaesthesia Korle Bu Teaching Hospital Accra Ghana; 8 Chemical Pathology Unit, Department of Medical Laboratory Sciences School of Biomedical and Allied Health Sciences University of Ghana Accra Ghana; 9 National Diabetes Management and Research Centre Korle Bu Teaching Hospital Accra Ghana; 10 Department of Preventive and Community Dentistry University of Ghana Dental School Accra Ghana; 11 Eye Department Cape Coast Teaching Hospital Accra Ghana; 12 Ophthalmology and Visual Sciences (DCN) Faculty of Medicine and Health Sciences, School of Clinical Sciences University Hospital Nottingham United Kingdom

**Keywords:** diabetes mellitus, type 1 diabetes, type 2 diabetes, microvascular, macrovascular complications, quality of life

## Abstract

**Background:**

Diabetes is associated with premature morbidity and mortality from its many complications. There are limited data on the chronic complications of diabetes in children and adolescents in sub-Saharan Africa.

**Objective:**

The study aims to determine the (1) burden and related factors of chronic systemic complications of diabetes, including diabetic and nondiabetic ocular conditions in children and adolescents, and (2) quality of life (QoL) of participants compared to healthy controls. This manuscript describes the study methodology.

**Methods:**

Demographic information, medical history, anthropometric measurements, and laboratory characteristics were collected, and the participants were screened for microvascular and macrovascular complications as well as nondiabetic ocular disease. QoL questionnaires were administered to participants, their caregivers, and controls. Participants were followed up annually up to 3 years to determine the natural history of and trends in these conditions. SPSS Version 25.0 will be used for data analysis. Continuous and categorical data will be presented as mean (SD) and as percentages (%), respectively. t tests and analysis of variance will be used to compare means, and chi-square tests will be used to compare categorical data. Correlation, regression, and logistic regression analyses will be employed to establish linear associations and causal associations as appropriate. Relative risk and odds ratios will be used to estimate risk. QoL outcomes in Ghanaian children and adolescents with diabetes mellitus compared with caregivers and healthy controls will be assessed using the Pediatric Quality of Life inventory. Significance will be set at α=.05.

**Results:**

Institutional approval from the Ethical and Protocol Review Committee of the University of Ghana Medical School was received on August 22, 2014 (Protocol Identification Number: MS-Et/M.12-P4.5/2013-2014). Funding for the project was received from the University of Ghana Research Fund (#UGRF/9/LMG-013/2015-2016) in March 2016. Patient recruitment, clinical examination, and data collection commenced in August 2016 and was completed in September 2019. A total of 58 children and adolescents with diabetes mellitus have been recruited. Blood samples were stored at –80 °C for analysis, which was completed at the end of July 2020. Data analysis is ongoing and will be completed by the end of December 2020. Investigators plan to submit the results for publication by the end of February 2021.

**Conclusions:**

The prevalence, natural history, trends in diabetic complications and nondiabetic ocular disease, and QoL will be provided. Our data may inform policies and interventions to improve care given to children and adolescents with diabetes.

**International Registered Report Identifier (IRRID):**

DERR1-10.2196/21440

## Introduction

Diabetes mellitus (DM), in general, is a challenge for all ages, as it leads to significant morbidity, especially kidney, eye, heart, and cerebrovascular disease, and premature mortality [1,2]. There are limited data on the epidemiology of DM in different sub-Saharan African (SSA) populations [3-6]. Danquah et al [7] reported the prevalence of type 2 DM as 6% among Ghanaian adults in an urban area. Notably, almost all the existing epidemiological data on DM pertain to adults [4-6]. The limited data available in children are relatively old [8-13], and studies have reported prevalence rates of 10.1 per 100,000 [9] to 0.33 per 1000 in a Nigerian hospital [14] for children younger than 15 years. However, considering the life expectancy of these persons, these rates are very high. The rapid epidemiological shift being experienced by western countries [15,16], where overweight and obese children and adolescents are increasingly being diagnosed with type 2 DM, may possibly also manifest in SSA. DM predisposes to the development of generalized microangiopathy, with clinical consequences affecting the kidneys, eyes, and nerves, and macrovascular disease in children and adolescents [15,17,18]. Populations of African origin have been reported to have the highest prevalence of microvascular complications of DM [19]. Type 1 DM is one of the most frequent chronic diseases in children and represents a public health challenge globally [20,21]. Reported estimates for the prevalence of nephropathy, retinopathy, and poor growth range between 10% and 33%, whilst hemoglobin (Hb)A1c levels averaged 7.5%-12.5% for children and adolescents in the western world [22-24]. However, there are limited data available for SSA.

Although children and adolescents with type 1 DM are faced with the threat of acute complications of hypoglycemia and ketoacidosis on a daily basis, in the long-term, the microvascular and macrovascular complications of the disease place them at greatest risk for serious morbidity and earlier-than-expected mortality [25]. The burden of both type 1 and 2 DM among Ghanaian children and adolescents and their attendant chronic complications have not been previously documented. Further, nondiabetic eye conditions have not been studied in Ghanaian children with DM. There is also a lack of information on the natural history of diabetic complications in Ghanaian children or how these can be modified in the Ghanaian population. Currently, there is no national policy for the management of DM in Ghanaian children and adolescents.

It is known that clinical characteristics of type 1 and 2 DM in people from SSA may differ somewhat that in typical European populations [26]. Diabetic nephropathy and diabetic retinopathy (DR) are the leading causes of end-stage renal disease and blindness, respectively, in younger patients globally and pose major public health challenges. DM in children and adolescents is becoming a health problem in developing countries. Limited clinic-based data in Ghana suggest that the prevalence of childhood and adolescent DM is on the increase, a trend similar to that in ethnic minority populations in western countries [26]. To the best of our knowledge, there are limited or no data on the occurrence of ocular and kidney disease in children with DM in SSA including Ghana. There are no previous studies on the choroid and retina in these children.

It has been suggested that the age of onset of type 1 DM is later in African communities (22-29 years) including Tanzania (15-19 years), South Africa (21-30 years), and Ethiopia (20-25 years) compared to European populations [27]. Such an epidemiological shift from what occurs in the West is difficult to establish in Ghana currently, due to scarce data availability on the prevalence of type 1 or 2 DM in younger persons (<20 years).

DR is one of the long-term microvascular complications of DM in children and leads to debilitating effects on visual acuity and ultimately blindness in some cases [1,2]. The risk for development of DR is said to be variable but may be present at time of diagnosis of DM. The variability of the risk of retinopathy is corroborated by population-based studies from Australia and Sweden, where 24% [15] and 27% [28] of children and adolescents with type 1 DM develop retinopathy 6 and 13 years after DM diagnosis, respectively. The incidence of DR is dependent on age of diagnosis and duration [15,17]. Overall, DR affects 15%-55% of patients, with a high proportion of proliferative retinopathy and macular edema in type 1 DM. In adults with type 2 DM, 21%-25% have retinopathy at diagnosis of diabetes compared with 9.5% of those with type 1 DM [19,29]. A nationwide Danish prospective cohort study of children and adolescents with type 1 DM followed up for 8 years investigated the effect of the prepubertal duration of DM on the development of early retinopathy and elevated albumin excretion rate (>20 µg/min) [22]. This Danish study reported the prevalence of any level of retinopathy to be 17.7% in the age group 12-15 years, 45.4% in the age group 16-20 years, and increased to 67.6% after 20 years of age [22]. DR was significantly associated with poor long-term glycemic control (hemoglobin [Hb]A1c; *P*<.0001) and with DM duration in patients with either a prepubertal or pubertal onset of disease (*P*<.001). Prepubertal DM duration is significantly associated with the development of DR [22].

There are also significant nonvascular diabetic eye morbidities as younger DM patients are known to have increased risks of developing neurotrophic keratitis, cataracts, and cranial nerve palsies and may have refractive abnormalities, while adults may also develop retinal vascular occlusions [2,30-33].

This study aims to determine the burden of microvascular and macrovascular complications of diabetic and nondiabetic ocular conditions in Ghanaian children with DM and further, to evaluate different factors that may influence these complications or associations. Specifically, the study will:

1. Determine the hospital-based prevalence, incidence, onset, and trend of microvascular and macrovascular disease (retinopathy, nephropathy, and neuropathy; cardiovascular disease), as well as other ocular changes of diabetic (cataracts, corneal ulceration) and nondiabetic ocular disease in children and adolescents in Ghana.
2. Determine the relationship between the prevalence of these microvascular and macrovascular complications with age at diagnosis and duration of DM, HbA1c, lipid profile, and other inflammatory markers.
3. Determine differences in the patterns of presentation of microvascular and macrovascular complications with nutritional status (BMI-age-sex percentile or z-score).
4. Determine the clinical, social, and biochemical determinants of ocular, neuropathic, nephropathic, and cardiovascular complications of juvenile DM.
5. Investigate the quality of life (QoL) of children and adolescents living with DM compared to healthy controls and the perspectives of their carers.

## Methods

A multidisciplinary team of researchers from Europe and Ghana is assembled to achieve the study goals. These researchers have diverse experiences in clinic-based and population-based research in DM and other chronic disease in both adults and children.

### Study Design

The study was done in 2 phases comprising an initial cross-sectional study to determine the prevalence and characteristics of microvascular and macrovascular complications of juvenile DM as well as nondiabetic ocular diseases among participants over a 3-year period. QoL, risks, and interaction with other diseases including sickle cell disease or trait will be evaluated. The second phase was a longitudinal study in which participants from the first phase were followed up annually for 3 years (with possible extension to 5-10 years, subject to funding) to determine the natural history of the studied conditions.

### Study Site

The study was conducted at the outpatient clinics of the Departments of Child Health, Medicine & Therapeutics, and Family Medicine; the Ophthalmology Unit; and the National Diabetes Management and Research Centre, all at Korle-Bu Teaching Hospital (KBTH), Accra, and Cape-Coast Teaching Hospital (CCTH) in the Central Region of Ghana. KBTH is the national referral hospital whereas CCTH serves mainly the Central, Western, and Western North regions of Ghana.

### Study Population

Children aged 4-19 years diagnosed with DM attending the outpatient clinics at the study sites who fulfil the inclusion criteria, their carers, and healthy controls consisting of children and adolescents from identified educational or religious facilities in Accra will be included.

### Case Definition

A case is defined as any child aged 4-19 years diagnosed with DM attending the KBTH or the CCTH. DM was diagnosed in a patient with classic symptoms and random plasma glucose ≥11.1 mmol/L. Type 1 DM was confirmed by the presence of glutamic acid decarboxylase (GAD) antibodies, insulinopenia, or low levels of C-peptide (stimulated C peptide values <0.6 pmol/mL), and type 2 DM was diagnosed by a negative test for type 1 DM–specific antibodies in association with elevated fasting insulin or C-peptide (stimulated C-peptide assay >0.6 pmol/mL) and the presence of acanthosis nigricans [34-36].

### Inclusion Criteria

For the children with DM, all patients aged 4-19 years with DM (ie, with classic symptoms of hyperglycemia or hyperglycemic crisis, random plasma glucose ≥200 mg/dL [11.1 mmol/L]) who consented, assented, or whose carers consented for inclusion were included. For the healthy controls, children without DM, any form of chronic and psychiatric diseases, or coexisting acute disease during the study period were included.

### Exclusion Criteria

Patients aged 4-19 years with DM who did not or whose parents or guardians did not give consent for inclusion in the study were excluded. Patients with diabetes diagnosed before 4 years of age or after 19 years of age were also excluded. Finally, patients with chronic disease, psychiatric disease, or coexisting acute disease during the study period were excluded.

### Sampling

Children and adolescents with DM who met the inclusion criteria were recruited consecutively after written informed consent was given by their carers or themselves or after assent given by the children aged ≥8 years, as applicable. Healthy controls who met the inclusion criteria were also recruited conveniently after consent and assent, as applicable.

### Sample Size

The population of children and adolescents attending clinics at the study sites was expected to be low. As such, all available patients were recruited into the study. It was estimated from clinical data available from the proposed study sites that about 5-10 children were diagnosed with DM annually. From these available data, with a power of 80% at a significance level of .05, a sample size of at least 55 children was anticipated in this study. During the period of data collection, a total of 58 children with DM were recruited. A total of 80 age-matched healthy controls were recruited for the QoL outcomes study.

### Study Duration

An initial cross-sectional study was conducted in 2016 with follow-up in a longitudinal study over a period of 3 years (August 2016 to September 2019).

### Procedures

#### General

Written informed consent or assent (see Multimedia Appendix 1) was obtained from all subjects and their guardians. This was followed by the administration of predesigned structured questionnaires (see Multimedia Appendix 2) to collect all clinical data for the children and adolescents with DM. These included patient demography, past medical history, type of DM (type 1 or 2), features of any systemic complications (nephropathy, cardiac, neurological, fatty liver, peripheral vascular disease, leg ulcers, and other comorbid conditions recorded from case notes where available), and laboratory investigations (eg, blood glucose and HbA1c at diagnosis and during follow-up).

Blood pressure measurements and examination of all systems were recorded. Hb levels as well as Hb genotype status were determined by Hb electrophoresis.

#### Ocular

Ocular examination included visual acuity assessment using the appropriate LogMAR test type for age, anterior segment assessment using slit lamp binocular microscope, dilated fundoscopy with binocular indirect ophthalmoscopy and a 20D or 28D lens, and fundus biomicroscopy using a 78D or 90D lens. Ocular ultrasound (B mode) examination of the posterior segment of the eye with a Tomey UD 1000 Model (Tomey, Germany) was undertaken where media opacity precludes fundus visualization. Ocular fundus photography acquiring 4-field stereocolor photos from each eye with a VISUSCOUT 100 portable retina camera system was done. All retinal images were graded independently (according to the Early Treatment Diabetic Retinopathy Study [ETDRS]) by WMA and VAE, with joint adjudication when there were discrepancies. The ophthalmic examination and investigations were repeated at every visit (at 12-month intervals).

#### Systemic, Microvascular, and Macrovascular Functional Evaluation

A full physical examination was undertaken. Height and weight were measured, BMI was calculated in kg/m^2^, and BMI-age-sex z scores and percentiles were determined using Centers for Disease Control reference standards and Anthro Plus software.

Assessments of macrovascular and microvascular disease were performed at baseline and annually in all study subjects. Ankle brachial index (ABI) was measured according to the American Heart Association guidelines [37]. Huntleigh Doppler equipment with an 8-MHz Doppler probe (Huntleigh, UK) was used to determine flow reappearance during slow deflation of a blood pressure cuff. The systolic pressure of the posterior tibial and dorsalis pedis was measured in both ankles, and the higher of the 2 was used as the ABI numerator. The denominator was determined by the higher of the 2 systolic blood pressure readings, 1 taken from each arm. An ABI ≤0.90 was indicative of peripheral arterial disease. Assessment of coronary artery disease using a resting 12-lead electrocardiogram (Nova PC-based ECG system) was done and analyzed using the Minnesota Code. Clinical assessment of neuropathy included a physical examination as well as an assessment of vibration perception threshold (VPT) as recommended by the American Diabetes Association [38]. VPT was assessed using a hand-held neurothesiometer (Horwell Neurothesiometer, Scientific Laboratory Supplies Ltd, Nottingham, UK) according to the manufacturer’s guidelines. VPT was assessed at the metatarsophalangeal joint of both feet in a 2-step manner starting from 0 V with increasing stimulation and then starting from 50 V with decreasing stimulation.

#### Laboratory Investigations

Laboratory assessments were performed at the Diabetes Research and Chronic Disease Reference Laboratory, University of Ghana Medical School, and MDS-Lancet Laboratories Ghana Limited in Accra. Samples of serum and plasma aliquoted from appropriate collection tubes or whole blood from the collaborating regional hospitals were transported to the Diabetes Research and Chronic Disease Reference Laboratory on dry ice within 12 hours after sample acquisition and processed immediately or stored at –80 °C for later analysis. Type 1 DM was confirmed by the presence of GAD antibodies, insulinopenia, or low levels of C-peptide, and type 2 DM was diagnosed by a negative test for type 1 DM–specific antibodies in association with elevated fasting insulin or C-peptide and the presence of acanthosis nigricans. Blood C-peptide levels were determined by competitive enzyme immunoassay methods using ELISA kits (NovaTec Immundiagnostica GmbH, Dietzenbach, Germany). Islet cell antibodies were assayed using ICA Enzyme Immuno Assay Kits. Furthermore, GAD65 autoantibodies were assayed using another ELISA kit (Medizym anti-GAD Testkit 96 tests, Medipan GmbH, Blankenfelde-Mahlow, Germany). Presence and levels of thyroid antibodies were determined using a chemiluminescent microparticle immunoassay (Abbott Alinity, Abbott Park, IL). HbA1c was measured using the Tri-Stat Boronate Affinity System (Trinity Biotech, Ireland). Lipid profile was assayed by determining the total serum cholesterol (cholesterol oxidase, esterase), triglycerides (enzymatic end point), high-density lipoprotein cholesterol (direct measure polymer-polyanion), and low-density lipoprotein cholesterol by enzymatic methods using an automated chemistry analyzer (AU480 Beckman Coulter, Brea, CA). Nephropathy was assessed by the measurement of albuminuria using immunochromatography on fresh spot urine, and serum beta 2 macroglobulin was assessed by an immunoassay method (Siemens Immulite, Washington DC). Blood urea (urease UV method), electrolytes (ion selective electrodes [ISE] and enzymatic method for bicarbonates), and creatinine (Jaffe IDMS-traceable method) were assayed with the Beckman Coulter AU480. Hb electrophoresis was performed to determine the Hb type using the cellulose acetate paper method (tris-EDTA boric acid [TEB] buffer, pH 8.4). Urinalysis and microscopic examination of urine deposits were performed. Hematological examination included a complete blood count using a 3-part Mindray hematology autoanalyzer (Shanghai, China).

#### Other Investigations

Abdominal ultrasound (B mode) was performed on patients found to have abdominal masses on clinical examination at baseline then yearly using a Hitachi EUB-7500 (Japan-made, 2009-2010). Where there was a pathology detected on ultrasound, magnetic resonance imaging was performed with the Hitachi Airis Elite (Ibaraki, Japan).

#### Quality of Life Outcome

Children and adolescents with DM and healthy controls completed the Pediatric Quality of Life Inventory (PedsQL, Mapi Research Trust, Lyon, France; Multimedia Appendix 3). In addition, children and adolescents with DM completed the disease-specific (diabetes) module. Parents and carers also completed the proxy reports for the PedsQL 4.0 Generic Core Scales and the disease-specific (diabetes) modules, the PedsQL 2.0 Family Impact Module, and the PedsQL Family Information Form. Permission was sought from Mapi Research Trust for the use of the inventories (Multimedia Appendix 4). Each questionnaire took approximately 5-10 minutes to complete.

The PedsQL 4.0 Generic Core Scales has a set of questions for the participants and their parents or carers and healthy controls. For the participants and controls, the PedsQL asks questions about how they felt and what they thought about their health. For the parent, the PedsQL assesses the health-related QoL (HRQoL) of their children and adolescents. It contains questions about the child’s physical, emotional, social, and school functioning in the past 1 month. It is a 5-point Likert scale ranging from 0 (Never) to 4 (Almost Always) without weighting of items on the scale.

The PedsQL 3.2 Diabetes Module is a 33-item diabetes-specific, HRQoL instrument that is made up of 5 scales measuring diabetes symptoms (15 items), treatment barriers (5 items), treatment adherence (6 items), worry (3 items), and communication (4 items). The scale is made up of patient self-report and parent proxy report formats for ages 5-25 years and a parent proxy report format for ages 2-4 years, which analyzes the patient’s and parent’s perceptions of the patient’s diabetes-specific symptoms and management challenges. The patient self-report aspect was designed specifically for ages 5-7 years, 8-12 years, 13-18 years, and 18-25 years, while the parent proxy report forms are specific for ages 2-4 years (toddler), 5-7 years (young child), 8-12 years (child), 13-18 years (adolescent), and 18-25 years (young adult). The instrument seeks to know how much of a problem each item has been during the past 1 month. Items on the scale are reverse-scored and linearly transformed to a 0-100 scale (where 0=100, 1=75, 2=50, 3=25, 4=0). Lower scores indicate more diabetes symptoms and management problems, meaning lower diabetes-specific HRQoL. Summary scores are calculated as the sum of all the items divided by the number of items answered. When more than 50% of the items in the scale are missing, the summary score is not calculated.

The PedsQL 2.0 Family Impact Module is a 36-item tool that examines parents' self-reported HRQoL and family functioning due to their child’s health condition. The tool is made up of physical functioning (6 items), emotional functioning (5 items), social functioning (4 items), cognitive functioning (5 items), communication (3 items), worry (5 items), daily activities (3 items), and family relationships (5 items) of the parent or carer.

The PedsQL Family Information Form was completed by the parent or carers. The form is made up of the demographic characteristics of the child and parent and an impact scale assessment. Demographic characteristics such as age, sex, and ethnicity of the child and marital status, highest level of education, and occupation of both parents are documented. For the impact scale assessment, the presence of a chronic condition (defined as a physical or mental health condition that has lasted or is expected to last at least 6 months and interferes with the child’s activities) in the past 6 months, any overnight visit to the hospital or an emergency room or urgent care in the past 12 months, absenteeism from school in the past 30 days due to physical or mental health conditions, number of days in the past 30 days the child was sick in bed or too ill to play and needed someone to care for them due to a health condition, and parent’s absenteeism from work due to child’s ill health were all documented.

### Preliminary or Pilot Data

Our anecdotal clinical experience is that the prevalence of childhood and adolescent DM is on the increase and that complications including DR and nephropathy may be severe. This is based on the collective experiences of the physicians and ophthalmologists involved in this project as well as national projections.

### Primary Outcome Measures

The primary outcomes are the prevalence of DM in the young (4-19 years old) and occurrence of organ-specific complications (ie, nephropathy, retinopathy, and neuropathy) in this population.

### Secondary Outcome Measures

Secondary outcomes include incidence of diabetic complications from baseline to the end of the second year of the study, QoL and type of comorbid conditions, weight and height (for nutritional status using BMI-age-sex percentile and z-score), Hb levels and genotypes, and duration and number of follow-ups in the study.

Disease progression will also be evaluated, including incidences of nephropathy, retinopathy, visual loss, neuropathy, and peripheral arterial disease. Changes in the macular, retinal, and choroidal thickness at onset of DR and with time will be assessed and correlated with other parameters.

### Treatment of Identified Cases

All children with diseases or complications requiring treatment were treated or will be treated by the relevant specialty using medication and other treatment modalities used and prescribed routinely in Ghana as per Ghana Health Service or Teaching Hospital treatment policies as well as those of the National Health Insurance Scheme. There is currently no national policy for the management of DM in Ghanaian children and adolescents. As such, international guidelines for the management of children and adolescents with DM were also referred to, as necessary, including the American Diabetes Association Guidelines [36] and the Diagnosis and Management Guideline for Diabetes (type 1 and type 2) in children and young people (NICE, NG18) [39].

### Follow-Up of Patients in the Longitudinal Study

Follow-up visits for participants without ocular complications were scheduled annually. For participants with ocular complications at baseline or diagnosed during annual follow-up visits, subsequent follow-up examinations were scheduled for every 6-12 months as determined by the type and severity of the findings.

### Study Status

Currently, patient recruitment, clinical examinations, and some laboratory analysis have been completed. Blood samples are stored at –80 °C for analysis. Data captured to date have been cleaned for analyses.

### Ethical Approval

This study was approved by the Ethical and Protocol Review Committee of the College of Health Sciences, University of Ghana (protocol identification number: MS-Et/M.12-P4.5/2013-2014).

### Data Analysis

Data captured using Microsoft Office Access will be cleaned for analysis. Analysis will be done using SPSS Version 25.0. Continuous numerical data will be presented as mean (SD) and categorical data as percentages (%). For continuous variables that may be skewed, median and interquartile ranges will be used for their summary. Data will be presented as frequency, proportions, and percentages either in tables or pie and bar charts as appropriate. Chi-square tests will be used to compare proportions and tests for independence or associations of conditions among the categories of patients. *t* tests and analyses of variance will be used to compare means where appropriate. Correlation analysis will be used to establish linear associations for scale and ordinal variables, while regression and logistic regression analyses will be employed to establish causal associations in the data. Relative risk and odds ratios will be used to estimate risk in the samples. Significance will be set at α=.05.

QoL outcomes in Ghanaian children and adolescents with DM compared with parents or carers and healthy controls will be assessed using the PedsQL 4.0 Generic Core Scales inventory. In scoring the process, items on the inventory will be reverse-scored and linearly transformed to a 0-100 scale as follows: 0=100, 1=75, 2=50, 3=25, 4=0. When >50% of the items in the scale are missing, the scale scores will not be computed. When ≥50% items are completed, the mean of the completed items in the scale will be computed by summing the items over the total number of items answered. The Psychosocial Health Summary Score will be obtained by the summation of the items over the number of items answered in the Emotional, Social, and School Functioning Scales. The total score will finally be obtained by summing all the items over the number of items answered on all the scales.

To examine parents' self-reported HRQoL and family functioning due to their children’s health condition, the PedsQL 2.0 Family Impact Module will be used. The overall total score will be obtained by averaging all 36 items. To obtain the HRQOL summary score of a parent, the average of the 20 items comprising the Physical, Emotional, Social, and Cognitive Functioning scales will be computed. The Family Functioning Summary score will be calculated by averaging the 8 items comprising the Daily Activities and Family Relationships scales. The items on the Family Impact Module will be reverse-scored and transformed linearly to a 0-100 scale where the higher scores will show better (a less negative impact of the child’s health on the parent or family) HRQoL.

## Results

Institutional approval from the Ethical and Protocol Review Committee of the University of Ghana Medical School was received on August 22, 2014 (protocol identification number: MS-Et/M.12-P4.5/2013-2014). Funding for the project was received from the University of Ghana Research Fund (#UGRF/9/LMG-013/2015-2016) in March 2016.

Patient recruitment, clinical examination, and data collection commenced in August 2017 and were completed in September 2019. A total of 58 children and adolescents with DM has been recruited. Blood samples are stored at –80 °C for analysis, which was completed at the end of July 2020.

Data analysis is ongoing and will be completed by the end of December 2020. Investigators plan to submit the results for publication by the end of February 2021.

## Discussion

The population of SSA is rapidly expanding. Similarly, the prevalence of DM is reported to be on the increase, increasing the risks associated with increased population growth compared to the risk reduction in higher-income economies [40]. This increase in DM prevalence is faster in low-income and middle-income populations than in high-income populations in all age groups [41].

Clinical studies in adults from South Africa [42], Tanzania [12], Ethiopia [43], and Ghana [7,44] reviewed by Hall et al [26] suggest that the characteristics of type 1 and 2 DM in people from SSA may differ somewhat from typical European populations. It is suggested that the present study, when completed, will help elucidate any such differences in childhood and adolescent DM in SSA including Ghana. Children and adolescents with type 1 DM are threatened by acute complications of hypoglycemia and ketoacidosis on a daily basis. However, the long-term microvascular and macrovascular complications of the disease place them at greatest risk for serious morbidity and early mortality [25].

Although it is estimated that the prevalence of DM in the adult population of Ghana is approximately 10%, no data are available on childhood and adolescent DM in Ghana. However, when DM is coupled with the number of years ahead of persons in childhood and adolescence, morbidity and the drain on the individuals, their families, and society become very immense and require quantification. In order to establish the importance of this growing problem, it is important to determine the hospital-based prevalence, incidence, onset, and trends of neuropathy, microvascular and macrovascular diseases (retinopathy, nephropathy, and neuropathy; cardiovascular disease), and other changes of diabetic (cataracts, corneal ulceration) and nondiabetic ocular disease in children and adolescents in Ghana. Potential estimates of hospitalization and mortality may also be gleaned from this study. This will serve as a preamble to further investigate the population prevalence and trend of DM in Ghana. To the best of our knowledge, there are no data on the QoL of children and adolescents with DM in SSA. This study, when completed, will provide some data to fill such a void. The associations with obesity, as well as QoL of people with DM and their carers, will be of immense importance in national and SSA planning of health education programs on DM and other childhood morbidities.

Furthermore, nondiabetic eye diseases have not been previously studied in Ghanaian children with DM. In addition, there is a lack of information on the natural history of DM complications in Ghanaian children or how these can be modified in the Ghanaian population. Currently, there is no national policy for the management of DM in Ghanaian children and adolescents. Data from this study would therefore lead to the development of policy briefs and guidelines that will be submitted to the Ministry of Health to help with the planning and implementation of effective policies for DM screening and the early detection of diabetic complications and nondiabetic eye disease in children, for improved care.

The study may also offer an opportunity for training and mentorship of resident doctors and biomedical scientists to augment in building capacity toward clinical care and research in children and adolescents with DM.

## Appendix

Multimedia Appendix 1 Participants informed consent form.

Multimedia Appendix 2 Study questionnaires.

Multimedia Appendix 3 PedsQL 4.0 questionnaires.

Multimedia Appendix 4 Permission to use PedsQL 4.0 inventory.

Multimedia Appendix 5 Ethical clearance letter.

## Abbreviations

ABI: ankle brachial index
CCTH: Cape-Coast Teaching Hospital
DM: diabetes mellitus
DR: diabetic retinopathy
ETDRS: Early Treatment Diabetic Retinopathy Study
GAD: glutamic acid decarboxylase
Hb: hemoglobin
HRQoL: health-related quality of life
KBTH: Korle-Bu Teaching Hospital
PedsQL: Pediatric Quality of Life Inventory
QoL: quality of life
SSA: sub-Saharan Africa
VPT: vibration perception threshold
