# Supplementary material for: Morbidity and Complications of Diabetes Mellitus in Children and Adolescents in Ghana: Protocol for a Longitudinal Study
Source: JMIR Res Protoc. 2021 Jan 6;10(1):e21440. doi: 10.2196/21440 (PMC7817364; doi:10.2196/21440)
Supplement: Multimedia Appendix 1 [file resprot_v10i1e21440_app1.docx]

| UNIVERSITY OF GHANA SCHOOL OF MEDICINE AND DENTISTRY  COLLEGE OF HEALTH SCIENCES  *Department of Surgery* |
| --- |


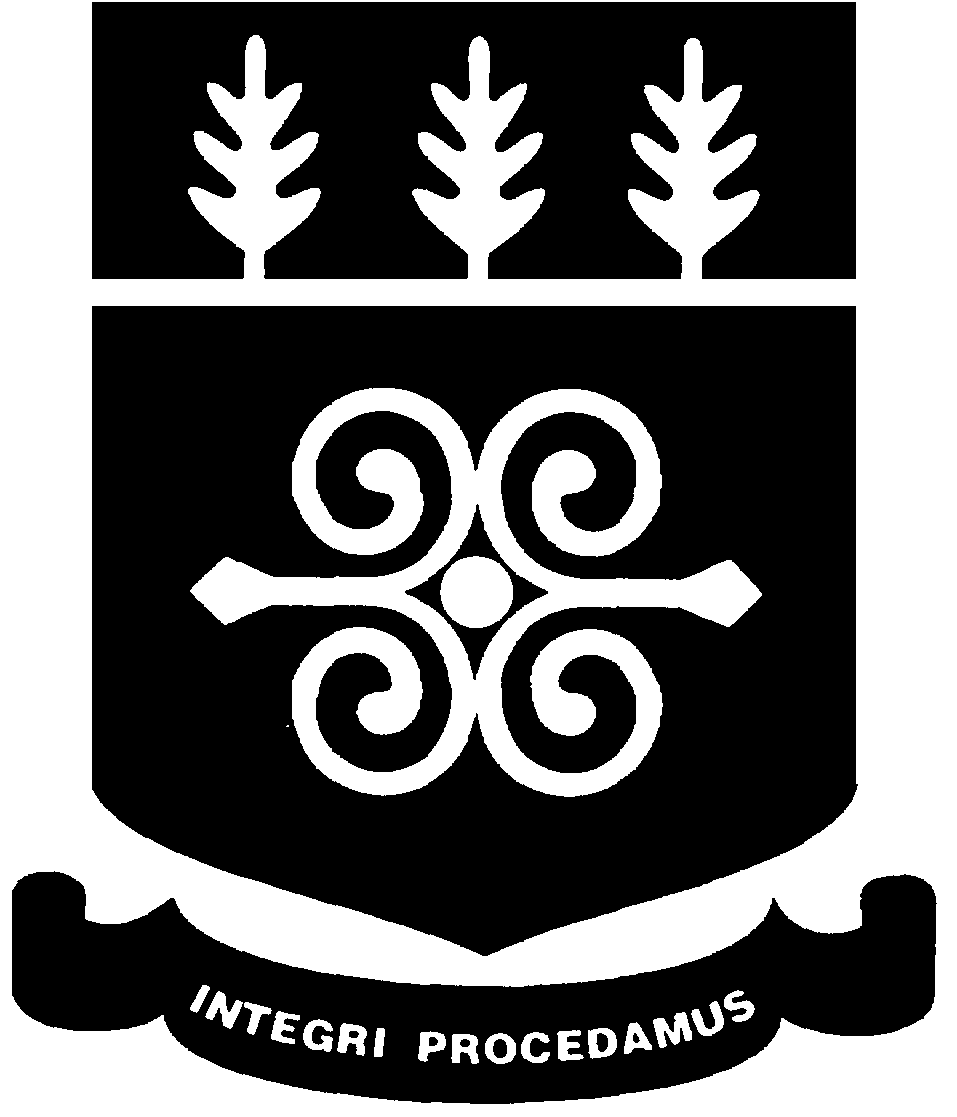


**APPENDIX 1A**

**INFORMED CONSENT FORM FOR PARTICIPANTS**

**Name of Principal Investigators**: Dr. V.A. Essuman, Prof. A.G.B. Amoah and Prof. W. Amoaku.

**Name of Institutions/ Departments:**

Department of Surgery, Department of Medicine, School of Medicine and Dentistry College of Health Sciences, University of Ghana. School of Medicine, University of Nottingham, United Kingdom.

**Project Title:**

The burden of complications of Diabetes Mellitus in Ghanaian children and adolescents.

**Ethics Approval ID**: MS-Et/M.12-P4.5/2013-2014

**Date:**...............................  **Study Site**....................................  **Patient Code**..............................

**Name of patient:**……………………………………….. **Age**................... **Gender**.........M/F

I...........................................................................................................have been invited to let my child/ward take part in the above-stated research.

**I have been told the purpose of this research is:** To establish the systemic and eye problems in children and adolescents with Diabetes Mellitus (DM).

Diabetes Mellitus is a condition which is associated with high blood glucose (sugar). The high blood glucose if not controlled over time may lead to complications that can result in poor eye sight or blindness, kidney damage, heart attack, stroke and amputation (loss of toe, foot or leg). Bringing down the high blood glucose reduces the occurrence of these complications. How often complications occur in Ghanaian children with Diabetes is not known.

Doctors and Scientists under the leadership of Dr. V.A. Essuman and Prof. A.G.B. Amoah ( from the University of Ghana School of Medicine and Dentistry) and Prof. W. Amoaku ( of the School of Medicine, University of Nottingham, United Kingdom) are therefore conducting a research to find out how common the above complications are in Ghanaian children aged 5 years to 19 years, at the Korle- Bu Teaching Hospital, Cape- Coast University Teaching Hospital and Effiah-Nkwanta Regional Hospital in Takoradi.

**I have been told the procedures of the study are**:

A complete examination including eye, general and laboratory examinations will be performed on me by a team of doctors and scientists. Consequently, I shall spend the best part of the morning at the health facility where I will be asked to provide certain information about me.

The eye examination will involve visual acuity assessment and instillation of dilating eye drops (Tropicamide and/or Phenylephrine) into my eyes to enable the doctors examine the back of my eyes (posterior segment).

The general examination will assess the whole body as is normally done.

Blood samples (5-15 mls at a time) will be taken from me at the beginning of the study and also during some of the follow up visits. This will help in performing special tests such as fasting lipids, HbA1c and others that will help with diagnosis and also assist in the monitoring of the DM and any associated complications that may be encountered. These tests will be at no cost to me or subsidised. Some of the blood samples may be stored for analysis later. Any future analysis of the blood samples will be done with prior approval from the Ethics Committee of the College of Health Sciences, University of Ghana. This amount of blood that will be taken is not very different from what I am normally asked to provide when I visit the diabetes clinic for my regular checkup. The amount of blood taken will not affect my health in any way.

I will be required to come for follow-up during the period of study once a year or earlier as determined by the type of complications encountered and also for some tests. I will be given some money for transport at each visit.

**The risks or dangers and discomforts are:**

I have been told that I may experience a minor bruise and/or temporary discomfort at the site of the blood drawn and this risk is no more than what I will normally be exposed to for having blood drawn routinely from the DM clinic.

The use of dilating eye drops may cause mild irritation of the eyes, transient blurring of vision and glare lasting between six and eight hours, which do not require treatment. I am advised to take extra care of myself until I regain my usual vision.

**The benefits are:**

I may benefit from the study by having thorough examinations performed on me, including eye examination, for early detection and management of complications associated with DM, some of which could cause irreversible blindness and possible death if not detected early. These examinations and treatment will be at no cost to me***.***

All test results will be explained to me. I may through this study discover certain complications in me which were previously unknown to me. If this is found to be the case, I will benefit from clinical counseling and be directed to the appropriate health facility for medical care.

The information obtained from the study would help to improve the care given to children and adolescents with DM in Ghana and also assist in suggesting ways of reducing complications in Ghanaian children with DM.

**Confidentiality:**

All information collected from this research will be kept confidential. The findings of this study may be reported at meetings or in medical journals, but my name and other personal identifiers will not be used in the report.

**My right to refuse or withdraw:**

I do not have to take part in this research if I do not wish to do so, and this will not affect my treatment at the clinic. I may stop participating in this research at any time that I wish to without losing any rights of mine to health care.

**Who I may contact:**

I have been told that this proposal has been reviewed and approved by the University of Ghana Medical School Ethical and Protocol Review Committee (EPRC). This is the committee whose job is to ensure that research participants are protected from harm. If I wish to find out more about this committee, I may contact Prof. Andrew Adjei, Chairman of the University of Ghana College of Health Sciences’ EPRC.

**Contact Information:**

If I have any questions I may ask now or later. If I wish to ask questions later, I may contact any of the following:

Dr Vera Adobea Essuman

Ophthalmology unit, Department of Surgery, School of Medicine and Dentistry,

College of Health Sciences, University of Ghana. Tel: 0244113337

Dr Josephine Akpaloo

Department of Medicine, School of Medicine and Dentistry,

College of Health Sciences, University of Ghana Tel: 0244622075

Mr. Benjamin Abaidoo

Ophthalmology unit, Department of Surgery, School of Medicine and Dentistry,

College of Health Sciences, University of Ghana.

Tel: 0277818746

I ……………………………………………… (*name of researcher/ or her associate*) have fully explained to …………………………………………… (*name of study participant*) the nature, purpose, procedures, risks and benefits associated with this study. I have answered and will answer to the best of my ability, all questions relating to the study.

………………………………………….. ………………… ………………….

***Full name of researcher or her associate Signature Date***

I …………………………….. (*name of study participant*) have read the foregoing information, or it has been translated to me in a language I understand. I have had the opportunity to ask questions about it and answers given me are to my satisfaction.

My signature or thumbprint below indicates that I have understood what is going to be done and I voluntarily agree to participate as a subject in this study.

Signed by......................................................................................................................................

Date ............................................ Place/ Study Site……………………………………..…….

If illiterate, signed by the investigator………………………………………………………….…

In the presence of an independent literate witness………………………………………………..

(where possible this person should be selected by the participant).

***Thumbprint (for the illiterate parent/ caretaker):***

Date…………………………. Place/ Study Site..…………………………………………...

| UNIVERSITY OF GHANA SCHOOL OF MEDICINE AND DENTISTRY  COLLEGE OF HEALTH SCIENCES  *Department of Surgery* |
| --- |


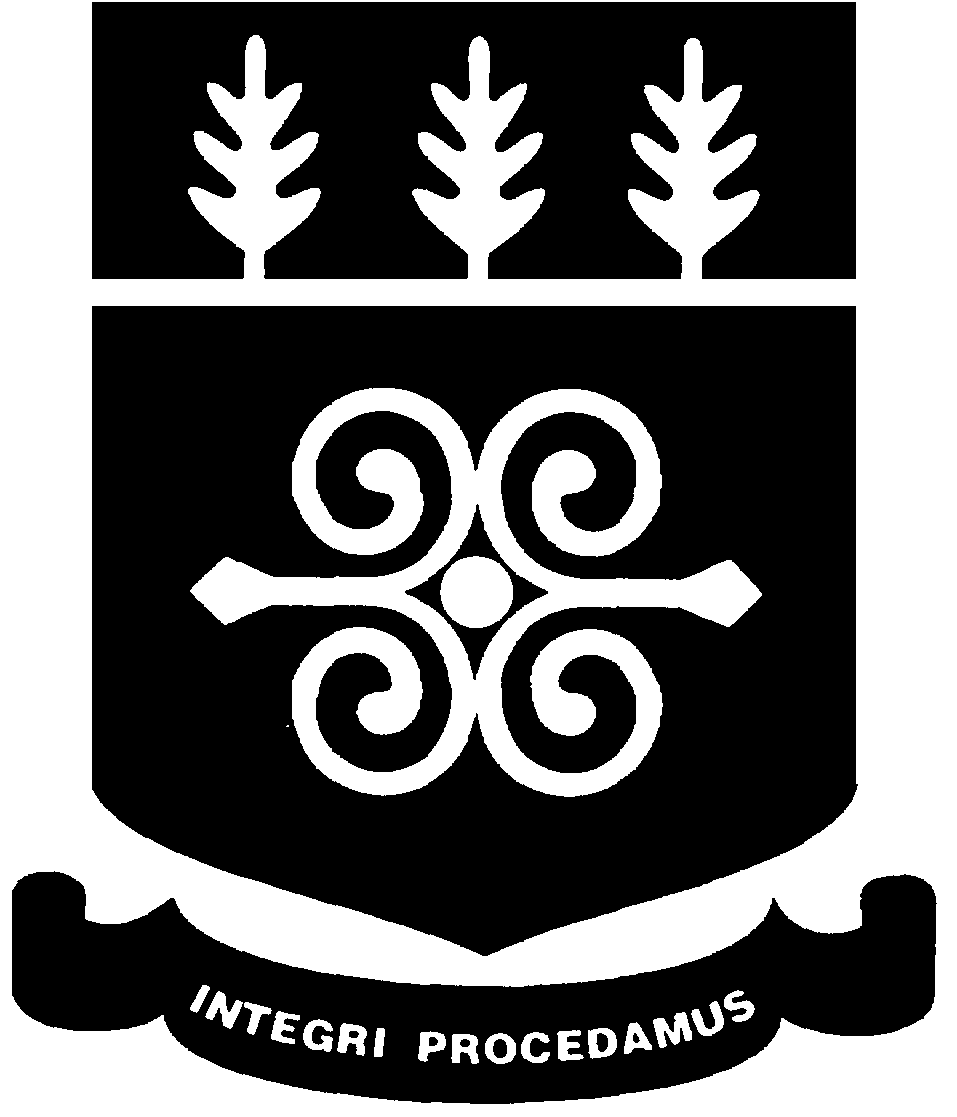


**APPENDIX 1B**

**INFORMED ASSENT FORM FOR MINOR PARTICIPANTS**

**Name of Principal Investigators**: Dr. V.A. Essuman, Prof. A.G.B. Amoah and Prof. W. Amoaku.

**Name of Institutions/ Departments:**

Department of Surgery, Department of Medicine, School of Medicine and Dentistry College of Health Sciences, University of Ghana. School of Medicine, University of Nottingham, United Kingdom.

**Project Title:**

The burden of complications of Diabetes Mellitus in Ghanaian children and adolescents.

**Ethics Approval ID**: MS-Et/M.12-P4.5/2013-2014

**Date:**...............................  **Study Site**....................................  **Patient Code**..............................

**Name of patient:**……………………………………….. **Age**................... **Gender**.........M/F

I...........................................................................................................have been invited to let my child/ward take part in the above-stated research.

**I have been told the purpose of this research is:** To establish the systemic and eye problems in children and adolescents with Diabetes Mellitus (DM).

Diabetes Mellitus is a condition which is associated with high blood glucose (sugar). The high blood glucose if not controlled over time may lead to complications that can result in poor eye sight or blindness, kidney damage, heart attack, stroke and amputation (loss of toe, foot or leg). Bringing down the high blood glucose reduces the occurrence of these complications. How often complications occur in Ghanaian children with Diabetes is not known.

Doctors and Scientists under the leadership of Dr. V.A. Essuman and Prof. A.G.B. Amoah ( from the University of Ghana School of Medicine and Dentistry) and Prof. W. Amoaku ( of the School of Medicine, University of Nottingham, United Kingdom) are therefore conducting a research to find out how common the above complications are in Ghanaian children aged 5 years to 19 years, at the Korle- Bu Teaching Hospital, Cape- Coast University Teaching Hospital and Effiah-Nkwanta Regional Hospital in Takoradi.

**I have been told the procedures of the study are**:

A complete examination including eye, general and laboratory examinations will be performed on me by a team of doctors and scientists. Consequently, I shall spend the best part of the morning at the health facility where my parents/guardians and I will be asked to provide certain information about me.

The eye examination will involve visual acuity assessment and instillation of dilating eye drops (Tropicamide and/or Phenylephrine) into my eyes to enable the doctors examine the back of my eyes (posterior segment).

The general examination will assess the whole body as is normally done.

Blood samples (5-15 mls at a time) will be taken from me at the beginning of the study and also during some of the follow up visits. This will help in performing special tests such as fasting lipids, HbA1c and others that will help with diagnosis and also assist in the monitoring of the DM and any associated complications that may be encountered. These tests will be at no cost to me or subsidised. Some of the blood samples may be stored for analysis later. Any future analysis of the blood samples will be done with prior approval from the Ethics Committee of the College of Health Sciences, University of Ghana. This amount of blood that will be taken is not very different from what I am normally asked to provide when we visit the diabetes clinic for my regular checkup. The amount of blood taken will not affect my health in any way.

My child and I will also be asked questions on quality of life issues relating to his/ her illness.

I will be required to come for follow-up during the period of study once a year or earlier as determined by the type of complications encountered and also for some tests. I will be given some money for transport at each visit.

**The risks or dangers and discomforts are:**

I have been told that I may experience a minor bruise and/or temporary discomfort at the site of the blood drawn and this risk is no more than what I will normally be exposed to for having blood drawn routinely from the DM clinic.

The use of dilating eye drops may cause mild irritation of the eyes, transient blurring of vision and glare lasting between six and eight hours, which do not require treatment. I am advised to take extra care of myself with the help of my parents/guardians until I regain my usual vision.

**The benefits are:**

I may benefit from the study by having thorough examinations performed on me, including eye examination, for early detection and management of complications associated with DM, some of which could cause irreversible blindness and possible death if not detected early. These examinations and treatment will be at no cost to me.

All test results will be explained to me and my parents/ guardians. I may through this study discover certain complications in me which were previously unknown to me. If this is found to be the case, I will benefit from clinical counseling and be directed to the appropriate health facility for medical care.

The information obtained from the study would help to improve the care given to children and adolescents with DM in Ghana and also assist in suggesting ways of reducing complications in Ghanaian children with DM.

**Confidentiality:**

All information collected from this research will be kept confidential. The findings of this study may be reported at meetings or in medical journals, but my name will not be used in the report.

**My right to refuse or withdraw:**

I do not have to take part in this research if I do not wish to do so, and this will not affect my treatment at the clinic. I may stop participating in this research at any time that I wish to without losing any rights of mine to health care.

**Who I may contact:**

I have been told that this proposal has been reviewed and approved by the University of Ghana Medical School Ethical and Protocol Review Committee (EPRC). This is the committee whose job is to ensure that research participants are protected from harm. If my parents/ guardians and I wish to find out more about this committee, we may contact Prof. Andrew Adjei, Chairman of the University of Ghana College of Health Sciences’ EPRC.

**Contact Information:**

If my parents/ guardians and I have any questions we may ask now or later. If we wish to ask questions later, we may contact any of the following:

Dr Vera Adobea Essuman

Ophthalmology unit, Department of Surgery, School of Medicine and Dentistry,

College of Health Sciences, University of Ghana. Tel: 0244113337

Dr Josephine Akpaloo

Department of Medicine, School of Medicine and Dentistry,

College of Health Sciences, University of Ghana Tel: 0244622075

Mr. Benjamin Abaidoo

Ophthalmology unit, Department of Surgery, School of Medicine and Dentistry,

College of Health Sciences, University of Ghana.

Tel: 0277818746

I ……………………………………………… (*name of researcher/ or her associate*) have fully explained to …………………………………………… (*name of study participant*) the nature, purpose, procedures, risks and benefits associated with this study. I have answered and will answer to the best of my ability, all questions relating to the study.

………………………………………….. ………………… ………………….

***Full name of researcher or her associate Signature Date***

I …………………………….. (*name of study participant*) have read the foregoing information, or it has been translated to me in a language I understand. I have had the opportunity to ask questions about it and answers given me are to my satisfaction.

My signature or thumbprint below indicates that I have understood what is going to be done and I voluntarily agree to participate as a subject in this study.

Signed by......................................................................................................................................

Date ............................................ Place/ Study Site……………………………………..…….

If illiterate, signed by the investigator………………………………………………………….…

In the presence of an independent literate witness………………………………………………..

(where possible this person should be selected by the participant).

***Thumbprint (for the illiterate parent/ caretaker):***

Date…………………………. Place/ Study Site..…………………………………………...

| UNIVERSITY OF GHANA SCHOOL OF MEDICINE AND DENTISTRY  COLLEGE OF HEALTH SCIENCES  *Department of Surgery* |
| --- |


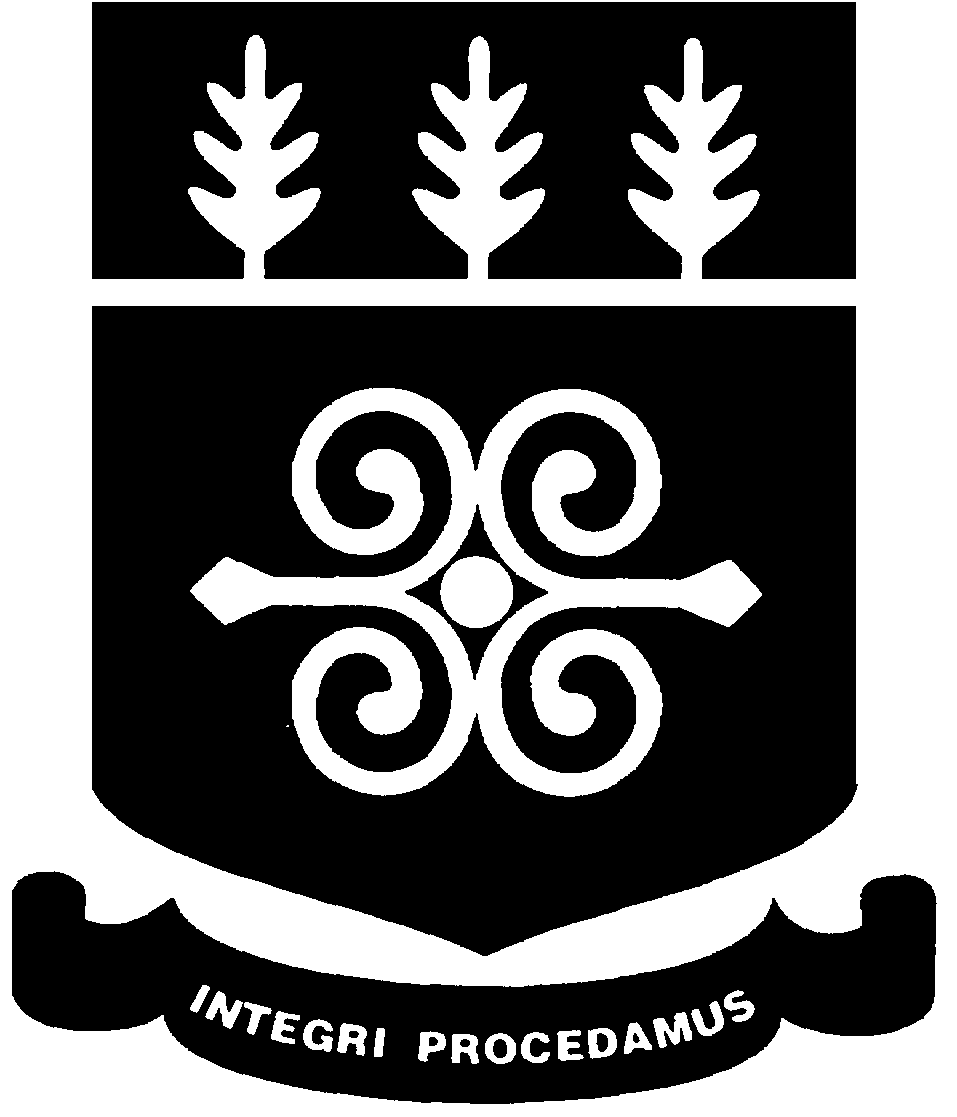


**APPENDIX 1C**

**INFORMED CONSENT FORM FOR PARENTS & CARE TAKERS OF MINORS**

**Name of Principal Investigators**: Dr. V.A. Essuman, Prof. A.G.B. Amoah and Prof. W. Amoaku.

**Name of Institutions/ Departments:**

Department of Surgery, Department of Medicine, School of Medicine and Dentistry College of Health Sciences, University of Ghana. School of Medicine, University of Nottingham, United Kingdom.

**Project Title:**

The burden of complications of Diabetes Mellitus in Ghanaian children and adolescents.

**Ethics Approval ID**: MS-Et/M.12-P4.5/2013-2014

**Date:**...............................  **Study Site**....................................  **Patient Code**..............................

**Name of patient:**……………………………………….. **Age**................... **Gender**.........M/F

I...................................................Parent/Guardian of ................................................................have been invited to let my child/ward take part in the above-stated research.

**I have been told the purpose of this research is:** To establish the systemic and eye problems in children and adolescents with Diabetes Mellitus (DM).

Diabetes Mellitus is a condition which is associated with high blood glucose (sugar). The high blood glucose if not controlled over time may lead to complications that can result in poor eye sight or blindness, kidney damage, heart attack, stroke and amputation (loss of toe, foot or leg). Bringing down the high blood glucose reduces the occurrence of these complications. How often complications occur in Ghanaian children with Diabetes is not known.

Doctors and Scientists under the leadership of Dr. V.A. Essuman and Prof. A.G.B. Amoah ( from the University of Ghana School of Medicine and Dentistry) and Prof. W. Amoaku ( of the School of Medicine, University of Nottingham, United Kingdom) are therefore conducting a research to find out how common the above complications are in Ghanaian children aged 5 years to 19 years, at the Korle- Bu Teaching Hospital, Cape- Coast University Teaching Hospital and Effiah-Nkwanta Regional Hospital in Takoradi.

**I have been told the procedures of the study are**:

A complete examination including eye, general and laboratory examinations will be performed on my child/ward by a team of doctors and scientists. Consequently, I shall spend the best part of the morning at the health facility where I will be asked to provide certain information about my child.

The eye examination will involve visual acuity assessment and instillation of dilating eye drops (Tropicamide and/or Phenylephrine) into my ward’s/child’s eyes to enable the doctors to examine the back of the eye (posterior segment).

The general examination will assess the whole body as is normally done.

Blood samples (5-15 mls at a time) will be taken from my child /ward at the beginning of the study and also during some of the follow up visits. This will help in performing special tests such as fasting lipids, HbA1c and others that will help with diagnosis and also assist in the monitoring of the DM and any associated complications that may be encountered. These tests will be at no cost to me or subsidised. Some of the blood samples may be stored for analysis later. Any future analyses of the blood samples will be done with prior approval from the Ethics Committee of the College of Health Sciences, University of Ghana. This amount of blood that will be taken is not very different from what my child is normally asked to provide when we visit the diabetes clinic for my child’s regular checkup. The amount of blood taken will not affect my child’s health in any way.

I will be required to bring my child/ ward for follow-up during the period of study once a year or earlier as determined by the type of complications encountered and also for some tests. I will be given some money for transport at each visit.

**The risks or dangers and discomforts are:**

I have been told that my child may experience a minor bruise and/or temporary discomfort at the site of the blood drawn and this risk is no more than what my child will normally be exposed to for having blood drawn routinely from the DM clinic.

The use of dilating eye drops may cause mild irritation of the eyes, transient blurring of vision and glare lasting between six and eight hours, which do not require treatment. I am advised to take extra care of my child and guide him / her until he /she regains usual vision.

**The benefits are:**

My child/ward may benefit from the study by having him/her examined thoroughly, including eye examination, for early detection and management of complications associated with DM, some of which could cause irreversible blindness and possible death if not detected early. These examinations and treatment will be at no cost to me.

All test results will be explained to me. I may through this study discover certain complications in my child/ward which were previously unknown to me. If this is found to be the case, I will benefit from clinical counseling and be directed to the appropriate health facility for medical care.

The information obtained from the study would help to improve the care given to children and adolescents with DM in Ghana and also assist in suggesting ways of reducing complications in Ghanaian children with DM.

**Confidentiality:**

All information collected from this research will be kept confidential. The findings of this study may be reported at meetings or in medical journals, but my child/ward’s name will not be used in the report.

**My right to refuse or withdraw:**

I do not have to let my child/ward take part in this research if I do not wish to do so, and this will not affect my child/ward’s treatment at the clinic. I may stop my child from participating in this research at any time that I wish to without losing any rights of my child/ward to health care.

**Who I may contact:**

I have been told that this proposal has been reviewed and approved by the University of Ghana Medical School Ethical and Protocol Review Committee (EPRC). This is the committee whose job is to ensure that research participants are protected from harm. If I wish to find out more about this committee, I may contact Prof. Andrew Adjei, Chairman of the University of Ghana College of Health Sciences’ EPRC.

**Contact Information:**

If I have any questions I may ask now or later. If I wish to ask questions later, I may contact any of the following:

Dr Vera Adobea Essuman

Ophthalmology unit, Department of Surgery, School of Medicine and Dentistry,

College of Health Sciences, University of Ghana. Tel: 0244113337

Dr Josephine Akpaloo

Department of Medicine, School of Medicine and Dentistry,

College of Health Sciences, University of Ghana Tel: 0244622075

Mr. Benjamin Abaidoo

Ophthalmology unit, Department of Surgery, School of Medicine and Dentistry,

College of Health Sciences, University of Ghana.

Tel: 0277818746

I ……………………………………………… (*name of researcher/ or her associate*) have fully explained to …………………………………………… (*name of study participant’s parent/ caretaker*) the nature, purpose, procedures, risks and benefits associated with this study. I have answered and will answer to the best of my ability, all questions relating to the study.

………………………………………….. ………………… ………………….

***Full name of researcher or her associate Signature Date***

I …………………………….. (*name of study participant’s parent/ caretaker*) have read the foregoing information, or it has been translated to me in a language I understand. I have had the opportunity to ask questions about it and answers given me are to my satisfaction.

My signature or thumbprint below indicates that I have understood what is going to be done and I consent voluntarily to allow my child/ward to participate as a subject in this study.

Signed by......................................................................................................................................

Date ............................................ Place/ Study Site……………………………………..…….

If illiterate, signed by the investigator………………………………………………………….…

In the presence of an independent literate witness………………………………………………..

(where possible this person should be selected by the participant).

***Thumbprint (for the illiterate parent/ caretaker):***

Date…………………………. Place/ Study Site..…………………………………………...
