# Supplementary material for: Morbidity and Complications of Diabetes Mellitus in Children and Adolescents in Ghana: Protocol for a Longitudinal Study
Source: JMIR Res Protoc. 2021 Jan 6;10(1):e21440. doi: 10.2196/21440 (PMC7817364; doi:10.2196/21440)
Supplement: Multimedia Appendix 2 [file resprot_v10i1e21440_app2.docx]

**APPENDIX 2**

**Morbidity Complications of Diabetes Mellitus in Ghanaian Children and Adolescents**

Date of examination: .............. ……………

Name of Doctor completing form: ………………………………………………

Study centre referred from: a. Child Health b. Medical OPD c. NDMRC d. Diabetes Youth Care e. Eye Centre f. Cape-Coast g. Effia- Nkwanta

1. **Identification / Demography**

1. Identification number………DM/00……………….

2. Folder number………………………………3. Age ……… Sex: male female

4. Ethnicity……………………………… 5. Educational level… ………………………

6. Date of diagnosis…………………………....7. Age at diagnosis.........................................

8. Weight (kg)...........................9.Height (m)...............................10. BMI......................................

11. Address: ……………………………………………………………………….........................

| 1. **History**   1. Presenting complaint  a. Ocular: .............................................................................................................................  ……………………………………………………………………………………………..  b. Systemic: ………………………………………………………………………………. ……………………………………………………………………………………………..  ……………………………………………………………………………………………..   1. **Systemic Evaluation**   2. Type of Diabetes Mellitus: a. Type I…… b. Type II……  3. Duration of DM...........................  4.Previous FBS recordings: a. Highest......................... b. Lowest.....................  5. HbA1c recordings: a. Previous / date: i…………...............................................  ii……………… …………………. iii…………………………………….……..  b. Current (baseline)……...................................................................  6. Type of treatment: (a) Insulin [Type/ dose……………………………………………]  (b) oral hypoglycaemics (c) combination  7. Presence of other co-morbid conditions   1. Hypertension 2. Asthma 3. Sickle cell disease 4. Hyperlipidaemias 5. Other (please specify) ..........................................   8. Immunization History (tick appropriately). Inspect Child Health Records if available.   1. Complete 2. Incomplete for age 3. Not done   9. Family History of Diabetes Mellitus? (circle appropriately)   1. Father (positive/negative) 2. Mother (positive/negative)   Other relation (please specify) ..................................  10. Number of siblings and Diabetes mellitus status   1. How many siblings............ 2. Is anyone a diabetic Yes/No 3. If yes, how many?........................   11. Family History of Thyroid disease? (circle appropriately)   1. Father (positive/negative) 2. Mother (positive/negative) 3. Other relation (please specify) ..................................   12. Family History of Eye diseases? (circle appropriately).   1. Father (positive/negative). Specify if positive………………………………… 2. Mother (positive/negative). Specify if positive……………………………….. 3. Other relation (please specify) ..................................   13. Other diabetic complications: Yes/No   \| 1. Diabetic foot \|  \| \| --- \| --- \| \| 1. Nephropathy \|  \| \| 1. Neuropathy – peripheral / autonomic \|  \| \| 1. Cardiac (*specify type):* \|  \| \| 1. Neurological (*e.g. cranial nerve palsies*) \|  \| \| 1. Fatty liver \|  \| \| 1. Peripheral vascular disease \|  \| \| 1. Hypoglycaemic episodes (specify dates) \|  \| \| 1. Diabetic ketoacidosis episodes (specify dates) \|  \| \| 1. Intercurrent illnesses (specify) \|  \| \| 1. Others *(please specify)* \|  \|  1. **Physical Examination** 2. **General**: Yes / No    1. Pallor______ b. Premature graying of hair_______   c. Vitiligo______ d. Hyperpigmentation_______ . e. Goitre_____  f. Acanthosis nigricans______ g. Pedal oedema_______   1. **Systems:**     CVS: a. Peripheral pulses: (i) present/weak/absent; (ii) rate_____     - 1. Blood Pressure: (i) Lying______ (ii) Standing_______   Abdomen (including liver span):  CNS – cranial nerves (by Ophthalmologists).   1. Motor: Muscle wasting (small muscles) ___________ 2. Reflexes (present/absent): Biceps (L/R) ________ Triceps (L/R) _________ 3. Clawing/deformities of feet ____________   Knee (L/R) _________ Ankle (L/R) _________   1. Sensation (present/absent): Vibration (L/R) ______ Fine touch (L/R)_________   Monofilament (L/R) _______ Pinprick (L/R) ________ Temp (L/R) __________   1. Specific Assessments: 2. Injection sites: 3. Cardiac assessment- Including ECG 4. Feet exam: absence of hair, dryness, cracks, calluses, warm/cold__________     Ulcers / location___________________________  Grade: superficial/deep/cellulitis/abscess/ bone involvement/gangrene  (local/general)   1. Ankle-Brachial Index [ABI]:   Doppler_________________ Digital sphygmomanometer_______________   1. Biothesiometry [peripheral nerve sensation] 2. Body Impedance Analysis [BIA] – for visceral fat measurement 3. **Laboratory Investigations**  \| **Test** \| **Results** \| \| \| \| --- \| --- \| --- \| --- \| \|  \| **Baseline** \| **Year 1** \| **Year 2** \| \| 1. FBC \|  \|  \|  \| \| 1. BUE&Cr \|  \|  \|  \| \| 1. Albumin / creatinine ratio \|  \|  \|  \| \| 1. C- peptide \|  \|  \|  \| \| 1. GAD -65 antibodies \|  \|  \|  \| \| 1. Hb Electrophoresis \|  \|  \|  \| \| 1. HbAIc [4 times a year] \| 1^st^ (Baseline):  2^nd^:  3^rd^:  4^th^: \|  \|  \| \| 1. Islet Cell antibodies \|  \|  \|  \| \| 1. Lipids- Triglycerides, cholesterol \|  \|  \|  \| \| 1. Thyroid antibodies \|  \|  \|  \| \| 1. Urinalysis [spot midstream urine specimen] \|  \|  \|  \| \| 1. serum Beta 2 macroglobulin \|  \|  \|  \| \| 1. Others:   Celiac - IgA ttg;  ab/anti-endomysial ab;  Abdominal USG.  a. Normal  b. Abnormal (please specify) \|  \|  \|  \|   **FOLLOW UP VISITS [GENERAL]**  **Date:**   1. **Identification / Demography**   1.Name of Patient:…………………………….Patient identification no.……………….  2. Folder number……………………………… 3. Age ……… Sex: male female   1. **History:** 2. **Physical Examination:**   **Baseline Ophthalmic Examination**  **Identification / Demography**   1. Patient name:………………………………………Identification no…DM/00……. 2. Folder number…………………………3. Age ……4. Sex male female 3. **Visual Acuity**  \| Visual acuity \| RE \| LE \| \| --- \| --- \| --- \| \| >6/18 \|  \|  \| \| 6/18->6/60 \|  \|  \| \| 6/60->3/60 \|  \|  \| \| <3/60 \|  \|  \| \| FFL \|  \|  \| |  |
| --- | --- | --- | --- | --- | --- | --- | --- | --- | --- | --- | --- | --- | --- | --- | --- | --- | --- | --- | --- | --- | --- | --- | --- | --- | --- | --- | --- | --- | --- | --- | --- | --- | --- | --- | --- | --- | --- | --- | --- | --- | --- | --- | --- | --- | --- | --- | --- | --- | --- | --- | --- | --- | --- | --- | --- | --- | --- | --- | --- | --- | --- | --- | --- | --- | --- | --- | --- | --- | --- | --- | --- | --- | --- | --- | --- | --- | --- | --- | --- | --- | --- | --- | --- | --- | --- | --- | --- | --- | --- | --- | --- | --- | --- | --- | --- | --- | --- | --- | --- | --- | --- |
| 1. Intraocular pressure (mmHg) RE…............ LE…............ 2. **Adnexal Manifestations**  \|  \| RIGHT EYE \| \| LEFT EYE \| \| \| --- \| --- \| --- \| --- \| --- \| \|  \| Yes \| No \| Yes \| No \| \| Blepharitis \|  \|  \|  \|  \| \| Allergic conjunctivitis \|  \|  \|  \|  \| \| Conjunctival growth \|  \|  \|  \|  \| \| Hordeoleum \|  \|  \|  \|  \| \| Recurrent (infectious) conjunctivitis \|  \|  \|  \|  \| \| Other (please specify \|  \|  \|  \|  \| |  |

1. **Anterior Segment Manifestations (please tick)**

|  | RIGHT EYE | | LEFT EYE | |
| --- | --- | --- | --- | --- |
|  | Yes | No | Yes | No |
| Dry eyes |  |  |  |  |
| TBUT |  |  |  |  |
| Corneal Arcus |  |  |  |  |
| Corneal ulcer:  Presumed bacterial  Presumed viral  Presumed fungal |  |  |  |  |
|  |  |  |  |  |
| Iris atrophy |  |  |  |  |
| Rubeosis iridis |  |  |  |  |
| RAPD |  |  |  |  |
| Uveitis:  Kp  Cells  Flare |  |  |  |  |
| Cataract |  |  |  |  |
| Type of cataract |  |  |  |  |
| - Cortical |  |  |  |  |
| - Nuclear |  |  |  |  |
| - Posterior subcapsular |  |  |  |  |
| - Other (please specify) |  |  |  |  |

1. **Posterior segment manifestations**

|  | RIGHT EYE | | LEFT EYE | | |
| --- | --- | --- | --- | --- | --- |
|  | Yes  INQ,SNQ,ITQ,STQ | No | | Yes  INQ,SNQ,ITQ,STQ | No |
| Vitreous cells |  |  | |  |  |
| Vitreous haemorrhage |  |  | |  |  |
| Cotton wool spots |  |  | |  |  |
|  | RIGHT EYE | | | LEFT EYE | |
|  | Yes  INQ,SNQ,ITQ,STQ | No | | Yes  INQ,SNQ,ITQ,STQ | No |
| Dot and blot haemorrhages |  |  | |  |  |
| Intraretinal haemorrhages |  |  | |  |  |
| Micro aneurysms |  |  | |  |  |
| Exudates |  |  | |  |  |
| Cystoid macula edema |  |  | |  |  |
| Fibrovascular membranes |  |  | |  |  |
| Tractional retinal detachment |  |  | |  |  |
| New vesssels on disc(NVD) |  |  | |  |  |
| New vessels elsewhere(NVE) |  |  | |  |  |
| NVE + NVD |  |  | |  |  |
| Optic disc swelling |  |  | |  |  |
| Optic atrophy |  |  | |  |  |
| Normal optic disc |  |  | |  |  |
| Others (specify)? |  |  | |  |  |
| Retinopathy type if present   - Non proliferative   - Mild   - Moderate   - Severe - Proliferative   - Non high risk   - High risk - CSMO |  |  | |  |  |

1. **Neurological examination**

Extraocular motility**:** Right eye Left eye

Anisocoria Yes  No

Cranial nerve evaluation (please tick (√) if normal and mark (x) if impaired)

|  | I | II | III | IV | V | VI | VII | VIII | IX | X | XI | XII |
| --- | --- | --- | --- | --- | --- | --- | --- | --- | --- | --- | --- | --- |
| OD |  |  |  |  |  |  |  |  |  |  |  |  |
| OS |  |  |  |  |  |  |  |  |  |  |  |  |

1. **Investigations**
2. Base line fundus photos:
3. Other Ocular test:
4. **Treatment Given**

| 1. Insulin | (please specify )…………………………………………… |
| --- | --- |
| 1. Oral hypoglycaemic drugs | (please specify) ……………………………………………............................... |
| 1. Combination | (please specify)…………………………………………….. |

**FOLLOW UP VISITS [OPHTHALMIC]**

**Identification / Demography**

1. Patient’s name:…………………………………………Identification no… DM/00…….….

2. Folder number……………………………… 3.Age ……… Sex male female

| \| **Number of Visits** \| **Visit #1** \| \| **Visit #2** \| \| **Visit # 3** \| \| **Visit # 4** \| \| \| --- \| --- \| --- \| --- \| --- \| --- \| --- \| --- \| --- \| \| Date \|  \| \|  \| \|  \| \|  \| \| \|  \| R \| L \| R \| L \| R \| L \| R \| L \| \| Visual acuity(unaided) \|  \|  \|  \|  \|  \|  \|  \|  \| \| Visual acuity (corrected) \|  \|  \|  \|  \|  \|  \|  \|  \| \| Intraocular pressure \|  \|  \|  \|  \|  \|  \|  \|  \| \| Blepharitis \|  \|  \|  \|  \|  \|  \|  \|  \| \| Allergic conjunctivitis \|  \|  \|  \|  \|  \|  \|  \|  \| \| Conjunctival growth \|  \|  \|  \|  \|  \|  \|  \|  \| \| Hordoleum \|  \|  \|  \|  \|  \|  \|  \|  \| \| Recurrent (infectious)conjunctivitis \|  \|  \|  \|  \|  \|  \|  \|  \| \| Other external eye signs (please specify) \|  \|  \|  \|  \|  \|  \|  \|  \| \| Dry eyes \|  \|  \|  \|  \|  \|  \|  \|  \| \| Corneal ulcer:  Presumed bacterial  Presumed viral  Presumed fungal \|  \|  \|  \|  \|  \|  \|  \|  \| \| Iris atrophy \|  \|  \|  \|  \|  \|  \|  \|  \| \| Rubeosis iridis \|  \|  \|  \|  \|  \|  \|  \|  \| \| RAPD \|  \|  \|  \|  \|  \|  \|  \|  \| \| Uveitis:  Kp  Cells  Flare \|  \|  \|  \|  \|  \|  \|  \|  \| \| cataract \|  \|  \|  \|  \|  \|  \|  \|  \| \| - cortical \|  \|  \|  \|  \|  \|  \|  \|  \| \| - nuclear \|  \|  \|  \|  \|  \|  \|  \|  \| \| - Posterior subcapsular \|  \|  \|  \|  \|  \|  \|  \|  \| \|  \| R \| L \| R \| L \| R \| L \| R \| L \| \| - Other (please specify) \|  \|  \|  \|  \|  \|  \|  \|  \| \| Vitreous cells \|  \|  \|  \|  \|  \|  \|  \|  \| \| Vitreous haemorrhage \|  \|  \|  \|  \|  \|  \|  \|  \| \| Cotton wool spots \|  \|  \|  \|  \|  \|  \|  \|  \| \| Dot and blot haemorrhages \|  \|  \|  \|  \|  \|  \|  \|  \| \| Intraretinal haemorrhages \|  \|  \|  \|  \|  \|  \|  \|  \| \| Micro aneurysms \|  \|  \|  \|  \|  \|  \|  \|  \| \| Exudates \|  \|  \|  \|  \|  \|  \|  \|  \| \| Cystoid macula edema \|  \|  \|  \|  \|  \|  \|  \|  \| \| Fibrovascular membranes \|  \|  \|  \|  \|  \|  \|  \|  \| \| Tractional retinal detachment \|  \|  \|  \|  \|  \|  \|  \|  \| \| New vesssels on disc(NVD) \|  \|  \|  \|  \|  \|  \|  \|  \| \| New vessels elsewhere(NVE) \|  \|  \|  \|  \|  \|  \|  \|  \| \| NVE + NVD \|  \|  \|  \|  \|  \|  \|  \|  \| \| Optic disc swelling \|  \|  \|  \|  \|  \|  \|  \|  \| \| Optic atrophy \|  \|  \|  \|  \|  \|  \|  \|  \| \| Normal optic disc \|  \|  \|  \|  \|  \|  \|  \|  \| \| Others (specify)? \|  \|  \|  \|  \|  \|  \|  \|  \| \| Retinopathy type if present   - Non proliferative   - Mild   - Moderate   - Severe - Proliferative   - Non high risk   - High risk - CSMO \|  \|  \|  \|  \|  \|  \|  \|  \| |  |
| --- | --- | --- | --- | --- | --- | --- | --- | --- | --- | --- | --- | --- | --- | --- | --- | --- | --- | --- | --- | --- | --- | --- | --- | --- | --- | --- | --- | --- | --- | --- | --- | --- | --- | --- | --- | --- | --- | --- | --- | --- | --- | --- | --- | --- | --- | --- | --- | --- | --- | --- | --- | --- | --- | --- | --- | --- | --- | --- | --- | --- | --- | --- | --- | --- | --- | --- | --- | --- | --- | --- | --- | --- | --- | --- | --- | --- | --- | --- | --- | --- | --- | --- | --- | --- | --- | --- | --- | --- | --- | --- | --- | --- | --- | --- | --- | --- | --- | --- | --- | --- | --- | --- | --- | --- | --- | --- | --- | --- | --- | --- | --- | --- | --- | --- | --- | --- | --- | --- | --- | --- | --- | --- | --- | --- | --- | --- | --- | --- | --- | --- | --- | --- | --- | --- | --- | --- | --- | --- | --- | --- | --- | --- | --- | --- | --- | --- | --- | --- | --- | --- | --- | --- | --- | --- | --- | --- | --- | --- | --- | --- | --- | --- | --- | --- | --- | --- | --- | --- | --- | --- | --- | --- | --- | --- | --- | --- | --- | --- | --- | --- | --- | --- | --- | --- | --- | --- | --- | --- | --- | --- | --- | --- | --- | --- | --- | --- | --- | --- | --- | --- | --- | --- | --- | --- | --- | --- | --- | --- | --- | --- | --- | --- | --- | --- | --- | --- | --- | --- | --- | --- | --- | --- | --- | --- | --- | --- | --- | --- | --- | --- | --- | --- | --- | --- | --- | --- | --- | --- | --- | --- | --- | --- | --- | --- | --- | --- | --- | --- | --- | --- | --- | --- | --- | --- | --- | --- | --- | --- | --- | --- | --- | --- | --- | --- | --- | --- | --- | --- | --- | --- | --- | --- | --- | --- | --- | --- | --- | --- | --- | --- | --- | --- | --- | --- | --- | --- | --- | --- | --- | --- | --- | --- | --- | --- | --- | --- | --- | --- | --- | --- | --- | --- | --- | --- | --- | --- | --- | --- | --- | --- | --- | --- | --- | --- | --- | --- | --- | --- | --- | --- | --- | --- | --- | --- | --- | --- | --- | --- | --- | --- | --- | --- | --- | --- | --- | --- | --- | --- | --- | --- | --- | --- | --- | --- | --- | --- | --- | --- | --- | --- | --- | --- | --- | --- | --- | --- | --- | --- | --- | --- | --- | --- | --- | --- | --- | --- | --- | --- | --- | --- | --- | --- | --- | --- | --- | --- | --- | --- | --- |

**Others:**
