# Supplementary material for: Morbidity and Complications of Diabetes Mellitus in Children and Adolescents in Ghana: Protocol for a Longitudinal Study
Source: JMIR Res Protoc. 2021 Jan 6;10(1):e21440. doi: 10.2196/21440 (PMC7817364; doi:10.2196/21440)
Supplement: Multimedia Appendix 3 [file resprot_v10i1e21440_app3.pdf]

ID# ___________________________

Date:___________________________

PedsQL™

Adult Quality of Life Inventory

Version 4.0

ADULT REPORT

DIRECTIONS

On the following page is a list of things that might be a problem for you. Please tell us **how much of a problem** each one has been for you during the **past ONE month** by circling:

0 if it is **never** a problem
1 if it is **almost never** a problem
2 if it is **sometimes** a problem
3 if it is **often** a problem
4 if it is **almost always** a problem

There are no right or wrong answers.
If you do not understand a question, please ask for help.

Not to be reproduced without permission

Copyright © 1998 JW Varni, Ph.D. All rights reserved

*In the past **ONE month**, how much of a **problem** has this been for you …*

**ABOUT MY HEALTH AND ACTIVITIES *(problems with…)***	**Never**	**Almost Never**	**Some-times**	**Often**	**Almost Always**
1. It is hard for me to walk more than one block	0	1	2	3	4
2. It is hard for me to run	0	1	2	3	4
3. It is hard for me to do sports activity or exercise	0	1	2	3	4
4. It is hard for me to lift something heavy	0	1	2	3	4
5. It is hard for me to take a bath or shower by myself	0	1	2	3	4
6. It is hard for me to do chores around the house	0	1	2	3	4
7. I hurt or ache	0	1	2	3	4
8. I have low energy	0	1	2	3	4

**ABOUT MY FEELINGS *(problems with…)***	**Never**	**Almost Never**	**Some-times**	**Often**	**Almost Always**
1. I feel afraid or scared	0	1	2	3	4
2. I feel sad or blue	0	1	2	3	4
3. I feel angry	0	1	2	3	4
4. I have trouble sleeping	0	1	2	3	4
5. I worry about what will happen to me	0	1	2	3	4

**HOW I GET ALONG WITH OTHERS *(problems with…)***	**Never**	**Almost Never**	**Some-times**	**Often**	**Almost Always**
1. I have trouble getting along with other adults	0	1	2	3	4
2. Other adults do not want to be my friend	0	1	2	3	4
3. Other adults tease me	0	1	2	3	4
4. I cannot do things that others my age can do	0	1	2	3	4
5. It is hard to keep up with my peers	0	1	2	3	4

**ABOUT MY WORK/STUDIES *(problems with…)***	**Never**	**Almost Never**	**Some-times**	**Often**	**Almost Always**
1. It is hard to pay attention at work or school	0	1	2	3	4
2. I forget things	0	1	2	3	4
3. I have trouble keeping up with my work or studies	0	1	2	3	4
4. I miss work or school because of not feeling well	0	1	2	3	4
5. I miss work or school to go to the doctor or hospital	0	1	2	3	4

 Not to be reproduced without permission Copyright © 1998 JW Varni, Ph.D. All rights reserved

ID#______________________

Date:____________________

PedsQL ™

Adult Quality of Life Inventory

Version 4.0

PARENT REPORT for **ADULTS**

DIRECTIONS

On the following page is a list of things that might be a problem for **your child**. Please tell us **how much of a problem** each one has been for **your child** during the **past ONE month** by circling:

0 if it is **never** a problem
1 if it is **almost never** a problem
2 if it is **sometimes** a problem
3 if it is **often** a problem
4 if it is **almost always** a problem

There are no right or wrong answers.
If you do not understand a question, please ask for help.

*In the past **ONE month,** how much of a **problem** has your child had with …*

**PHYSICAL FUNCTIONING *(problems with…)***	**Never**	**Almost Never**	**Some-times**	**Often**	**Almost Always**
1. Walking more than one block	0	1	2	3	4
2. Running	0	1	2	3	4
3. Participating in sports activity or exercise	0	1	2	3	4
4. Lifting something heavy	0	1	2	3	4
5. Taking a bath or shower by him or herself	0	1	2	3	4
6. Doing chores around the house	0	1	2	3	4
7. Having hurts or aches	0	1	2	3	4
8. Low energy level	0	1	2	3	4

**EMOTIONAL FUNCTIONING *(problems with…)***	**Never**	**Almost Never**	**Some-times**	**Often**	**Almost Always**
1. Feeling afraid or scared	0	1	2	3	4
2. Feeling sad or blue	0	1	2	3	4
3. Feeling angry	0	1	2	3	4
4. Trouble sleeping	0	1	2	3	4
5. Worrying about what will happen to him or her	0	1	2	3	4

**SOCIAL FUNCTIONING *(problems with…)***	**Never**	**Almost Never**	**Some-times**	**Often**	**Almost Always**
1. Getting along with other adults	0	1	2	3	4
2. Other adults not wanting to be his or her friend	0	1	2	3	4
3. Getting teased by other adults	0	1	2	3	4
4. Not able to do things that others his or her age can do	0	1	2	3	4
5. Keeping up with other adults	0	1	2	3	4

**WORK/STUDIES FUNCTIONING *(problems with…)***	**Never**	**Almost Never**	**Some-times**	**Often**	**Almost Always**
1. Paying attention at work or school	0	1	2	3	4
2. Forgetting things	0	1	2	3	4
3. Keeping up with work or studies	0	1	2	3	4
4. Missing work or school because of not feeling well	0	1	2	3	4
5. Missing work or school to go to the doctor or hospital	0	1	2	3	4

Not to be reproduced without permission Copyright © 1998 JW Varni, Ph.D. All rights reserved

ID# ____________________________

Date:___________________________

PedsQL™

Young Adult Quality of Life Inventory

Version 4.0

YOUNG ADULT REPORT (ages **18-25**)

DIRECTIONS

On the following page is a list of things that might be a problem for you. Please tell us **how much of a problem** each one has been for you during the **past ONE month** by circling:

0 if it is **never** a problem
1 if it is **almost never** a problem
2 if it is **sometimes** a problem
3 if it is **often** a problem
4 if it is **almost always** a problem

There are no right or wrong answers.
If you do not understand a question, please ask for help.

Not to be reproduced without permission

Copyright © 1998 JW Varni, Ph.D. All rights reserved

*In the past **ONE month**, how much of a **problem** has this been for you …*

**ABOUT MY HEALTH AND ACTIVITIES *(problems with…)***	Never	Almost Never	Some-times	Often	Almost Always
1. It is hard for me to walk more than one block	0	1	2	3	4
2. It is hard for me to run	0	1	2	3	4
3. It is hard for me to do sports activity or exercise	0	1	2	3	4
4. It is hard for me to lift something heavy	0	1	2	3	4
5. It is hard for me to take a bath or shower by myself	0	1	2	3	4
6. It is hard for me to do chores around the house	0	1	2	3	4
7. I hurt or ache	0	1	2	3	4
8. I have low energy	0	1	2	3	4

**ABOUT MY FEELINGS *(problems with…)***	Never	Almost Never	Some-times	Often	Almost Always
1. I feel afraid or scared	0	1	2	3	4
2. I feel sad or blue	0	1	2	3	4
3. I feel angry	0	1	2	3	4
4. I have trouble sleeping	0	1	2	3	4
5. I worry about what will happen to me	0	1	2	3	4

**HOW I GET ALONG WITH OTHERS *(problems with…)***	Never	Almost Never	Some-times	Often	Almost Always
1. I have trouble getting along with other young adults	0	1	2	3	4
2. Other young adults do not want to be my friend	0	1	2	3	4
3. Other young adults tease me	0	1	2	3	4
4. I cannot do things that others my age can do	0	1	2	3	4
5. It is hard to keep up with my peers	0	1	2	3	4

**ABOUT MY WORK/STUDIES *(problems with…)***	Never	Almost Never	Some-times	Often	Almost Always
1. It is hard to pay attention at work or school	0	1	2	3	4
2. I forget things	0	1	2	3	4
3. I have trouble keeping up with my work or studies	0	1	2	3	4
4. I miss work or school because of not feeling well	0	1	2	3	4
5. I miss work or school to go to the doctor or hospital	0	1	2	3	4

Not to be reproduced without permission
Copyright © 1998 JW Varni, Ph.D. All rights reserved

ID#___________________________

Date:_________________________

PedsQL ™

Young Adult Quality of Life Inventory

Version 4.0

PARENT REPORT for **YOUNG ADULTS** (ages **18-25**)

DIRECTIONS

On the following page is a list of things that might be a problem for **your child**. Please tell us **how much of a problem** each one has been for **your child** during the **past ONE month** by circling:

0 if it is **never** a problem
1 if it is **almost never** a problem
2 if it is **sometimes** a problem
3 if it is **often** a problem
4 if it is **almost always** a problem

There are no right or wrong answers.
If you do not understand a question, please ask for help.

 Not to be reproduced without permission Copyright © 1998 JW Varni, Ph.D. All rights reserved

*In the past **ONE month,** how much of a **problem** has your child had with …*

**PHYSICAL FUNCTIONING *(problems with…)***	**Never**	**Almost Never**	**Some-times**	**Often**	**Almost Always**
1. Walking more than one block	0	1	2	3	4
2. Running	0	1	2	3	4
3. Participating in sports activity or exercise	0	1	2	3	4
4. Lifting something heavy	0	1	2	3	4
5. Taking a bath or shower by him or herself	0	1	2	3	4
6. Doing chores around the house	0	1	2	3	4
7. Having hurts or aches	0	1	2	3	4
8. Low energy level	0	1	2	3	4

**EMOTIONAL FUNCTIONING *(problems with…)***	**Never**	**Almost Never**	**Some-times**	**Often**	**Almost Always**
1. Feeling afraid or scared	0	1	2	3	4
2. Feeling sad or blue	0	1	2	3	4
3. Feeling angry	0	1	2	3	4
4. Trouble sleeping	0	1	2	3	4
5. Worrying about what will happen to him or her	0	1	2	3	4

**SOCIAL FUNCTIONING *(problems with…)***	**Never**	**Almost Never**	**Some-times**	**Often**	**Almost Always**
1. Getting along with other young adults	0	1	2	3	4
2. Other young adults not wanting to be his or her friend	0	1	2	3	4
3. Getting teased by other young adults	0	1	2	3	4
4. Not able to do things that others his or her age can do	0	1	2	3	4
5. Keeping up with other young adults	0	1	2	3	4

**WORK/STUDIES FUNCTIONING *(problems with…)***	**Never**	**Almost Never**	**Some-times**	**Often**	**Almost Always**
1. Paying attention at work or school	0	1	2	3	4
2. Forgetting things	0	1	2	3	4
3. Keeping up with work or studies	0	1	2	3	4
4. Missing work or school because of not feeling well	0	1	2	3	4
5. Missing work or school to go to the doctor or hospital	0	1	2	3	4

Not to be reproduced without permission Copyright © 1998 JW Varni, Ph.D. All rights reserved

ID# ___________________________

Date:__________________________

PedsQL ™

Pediatric Quality of Life Inventory

Version 4.0

TEEN REPORT (ages **13-18**)

DIRECTIONS

On the following page is a list of things that might be a problem for you. Please tell us **how much of a problem** each one has been for you during the **past ONE month** by circling:

0 if it is **never** a problem
1 if it is **almost never** a problem
2 if it is **sometimes** a problem
3 if it is **often** a problem
4 if it is **almost always** a problem

There are no right or wrong answers.
If you do not understand a question, please ask for help.

Copyright © 1998 JW Varni, Ph.D. All rights reserved

*In the past **ONE month**, how much of a **problem** has this been for you …*

ABOUT MY HEALTH AND ACTIVITIES ***(problems with…)***	Never	Almost Never	Some-times	Often	Almost Always
1. It is hard for me to walk more than one block	0	1	2	3	4
2. It is hard for me to run	0	1	2	3	4
3. It is hard for me to do sports activity or exercise	0	1	2	3	4
4. It is hard for me to lift something heavy	0	1	2	3	4
5. It is hard for me to take a bath or shower by myself	0	1	2	3	4
6. It is hard for me to do chores around the house	0	1	2	3	4
7. I hurt or ache	0	1	2	3	4
8. I have low energy	0	1	2	3	4

ABOUT MY FEELINGS ***(problems with…)***	Never	Almost Never	Some-times	Often	Almost Always
1. I feel afraid or scared	0	1	2	3	4
2. I feel sad or blue	0	1	2	3	4
3. I feel angry	0	1	2	3	4
4. I have trouble sleeping	0	1	2	3	4
5. I worry about what will happen to me	0	1	2	3	4

HOW I GET ALONG WITH OTHERS ***(problems with…)***	Never	Almost Never	Some-times	Often	Almost Always
1. I have trouble getting along with other teens	0	1	2	3	4
2. Other teens do not want to be my friend	0	1	2	3	4
3. Other teens tease me	0	1	2	3	4
4. I cannot do things that other teens my age can do	0	1	2	3	4
5. It is hard to keep up with my peers	0	1	2	3	4

ABOUT SCHOOL ***(problems with…)***	Never	Almost Never	Some-times	Often	Almost Always
1. It is hard to pay attention in class	0	1	2	3	4
2. I forget things	0	1	2	3	4
3. I have trouble keeping up with my schoolwork	0	1	2	3	4
4. I miss school because of not feeling well	0	1	2	3	4
5. I miss school to go to the doctor or hospital	0	1	2	3	4

Not to be reproduced without permission

Copyright © 1998 JW Varni, Ph.D. All rights reserved

ID#__________________________

Date:_________________________

PedsQL ™

Pediatric Quality of Life Inventory

Version 4.0

PARENT REPORT for **TEENS** (ages **13-18**)

DIRECTIONS

On the following page is a list of things that might be a problem for **your teen**. Please tell us **how much of a problem** each one has been for **your teen** during the **past ONE month** by circling:

0 if it is **never** a problem
1 if it is **almost never** a problem
2 if it is **sometimes** a problem
3 if it is **often** a problem
4 if it is **almost always** a problem

There are no right or wrong answers.
If you do not understand a question, please ask for help.

 Copyright © 1998 JW Varni, Ph.D. All rights reserved

*In the past **ONE month,** how much of a **problem** has your teen had with …*

PHYSICAL FUNCTIONING ***(problems with…)***	Never	Almost Never	Some-times	Often	Almost Always
1. Walking more than one block	0	1	2	3	4
2. Running	0	1	2	3	4
3. Participating in sports activity or exercise	0	1	2	3	4
4. Lifting something heavy	0	1	2	3	4
5. Taking a bath or shower by him or herself	0	1	2	3	4
6. Doing chores around the house	0	1	2	3	4
7. Having hurts or aches	0	1	2	3	4
8. Low energy level	0	1	2	3	4

EMOTIONAL FUNCTIONING ***(problems with…)***	Never	Almost Never	Some-times	Often	Almost Always
1. Feeling afraid or scared	0	1	2	3	4
2. Feeling sad or blue	0	1	2	3	4
3. Feeling angry	0	1	2	3	4
4. Trouble sleeping	0	1	2	3	4
5. Worrying about what will happen to him or her	0	1	2	3	4

SOCIAL FUNCTIONING ***(problems with…)***	Never	Almost Never	Some-times	Often	Almost Always
1. Getting along with other teens	0	1	2	3	4
2. Other teens not wanting to be his or her friend	0	1	2	3	4
3. Getting teased by other teens	0	1	2	3	4
4. Not able to do things that other teens his or her age can do	0	1	2	3	4
5. Keeping up with other teens	0	1	2	3	4

SCHOOL FUNCTIONING ***(problems with…)***	Never	Almost Never	Some-times	Often	Almost Always
1. Paying attention in class	0	1	2	3	4
2. Forgetting things	0	1	2	3	4
3. Keeping up with schoolwork	0	1	2	3	4
4. Missing school because of not feeling well	0	1	2	3	4
5. Missing school to go to the doctor or hospital	0	1	2	3	4

 Not to be reproduced without permission Copyright © 1998 JW Varni, Ph.D. All rights reserved

ID# ___________________________

Date:___________________________

PedsQL ™

Pediatric Quality of Life Inventory

Version 4.0

CHILD REPORT (ages **8-12**)

DIRECTIONS

On the following page is a list of things that might be a problem for you. Please tell us **how much of a problem** each one has been for you during the **past ONE month** by circling:

0 if it is **never** a problem
1 if it is **almost never** a problem
2 if it is **sometimes** a problem
3 if it is **often** a problem
4 if it is **almost always** a problem

There are no right or wrong answers.
If you do not understand a question, please ask for help.

 Copyright © 1998 JW Varni, Ph.D. All rights reserved

*In the past **ONE month**, how much of a **problem** has this been for you …*

ABOUT MY HEALTH AND ACTIVITIES *(problems with…)*	Never	Almost Never	Some-times	Often	Almost Always
1. It is hard for me to walk more than one block	0	1	2	3	4
2. It is hard for me to run	0	1	2	3	4
3. It is hard for me to do sports activity or exercise	0	1	2	3	4
4. It is hard for me to lift something heavy	0	1	2	3	4
5. It is hard for me to take a bath or shower by myself	0	1	2	3	4
6. It is hard for me to do chores around the house	0	1	2	3	4
7. I hurt or ache	0	1	2	3	4
8. I have low energy	0	1	2	3	4

ABOUT MY FEELINGS *(problems with…)*	Never	Almost Never	Some-times	Often	Almost Always
1. I feel afraid or scared	0	1	2	3	4
2. I feel sad or blue	0	1	2	3	4
3. I feel angry	0	1	2	3	4
4. I have trouble sleeping	0	1	2	3	4
5. I worry about what will happen to me	0	1	2	3	4

HOW I GET ALONG WITH OTHERS *(problems with…)*	Never	Almost Never	Some-times	Often	Almost Always
1. I have trouble getting along with other kids	0	1	2	3	4
2. Other kids do not want to be my friend	0	1	2	3	4
3. Other kids tease me	0	1	2	3	4
4. I cannot do things that other kids my age can do	0	1	2	3	4
5. It is hard to keep up when I play with other kids	0	1	2	3	4

ABOUT SCHOOL *(problems with…)*	Never	Almost Never	Some-times	Often	Almost Always
1. It is hard to pay attention in class	0	1	2	3	4
2. I forget things	0	1	2	3	4
3. I have trouble keeping up with my schoolwork	0	1	2	3	4
4. I miss school because of not feeling well	0	1	2	3	4
5. I miss school to go to the doctor or hospital	0	1	2	3	4

Not to be reproduced without permission
Copyright © 1998 JW Varni, Ph.D. All rights reserved

ID# ___________________________

Date:__________________________

PedsQL ™

Pediatric Quality of Life Inventory

Version 4.0

PARENT REPORT for **CHILDREN** (ages **8-12**)

DIRECTIONS

On the following page is a list of things that might be a problem for **your child**. Please tell us **how much of a problem** each one has been for **your child** during the **past ONE month** by circling:

0 if it is **never** a problem
1 if it is **almost never** a problem
2 if it is **sometimes** a problem
3 if it is **often** a problem
4 if it is **almost always** a problem

There are no right or wrong answers.
If you do not understand a question, please ask for help.

Not to be reproduced without permission
Copyright © 1998 JW Varni, Ph.D. All rights reserved

*In the past **ONE month,** how much of a **problem** has your child had with …*

**PHYSICAL FUNCTIONING *(problems with…)***	**Never**	**Almost Never**	**Some-times**	**Often**	**Almost Always**
1. Walking more than one block	0	1	2	3	4
2. Running	0	1	2	3	4
3. Participating in sports activity or exercise	0	1	2	3	4
4. Lifting something heavy	0	1	2	3	4
5. Taking a bath or shower by him or herself	0	1	2	3	4
6. Doing chores around the house	0	1	2	3	4
7. Having hurts or aches	0	1	2	3	4
8. Low energy level	0	1	2	3	4

**EMOTIONAL FUNCTIONING *(problems with…)***	**Never**	**Almost Never**	**Some-times**	**Often**	**Almost Always**
1. Feeling afraid or scared	0	1	2	3	4
2. Feeling sad or blue	0	1	2	3	4
3. Feeling angry	0	1	2	3	4
4. Trouble sleeping	0	1	2	3	4
5. Worrying about what will happen to him or her	0	1	2	3	4

**SOCIAL FUNCTIONING *(problems with…)***	**Never**	**Almost Never**	**Some-times**	**Often**	**Almost Always**
1. Getting along with other children	0	1	2	3	4
2. Other kids not wanting to be his or her friend	0	1	2	3	4
3. Getting teased by other children	0	1	2	3	4
4. Not able to do things that other children his or her age can do	0	1	2	3	4
5. Keeping up when playing with other children	0	1	2	3	4

**SCHOOL FUNCTIONING *(problems with…)***	**Never**	**Almost Never**	**Some-times**	**Often**	**Almost Always**
1. Paying attention in class	0	1	2	3	4
2. Forgetting things	0	1	2	3	4
3. Keeping up with schoolwork	0	1	2	3	4
4. Missing school because of not feeling well	0	1	2	3	4
5. Missing school to go to the doctor or hospital	0	1	2	3	4

 Not to be reproduced without permission Copyright © 1998 JW Varni, Ph.D. All rights reserved

ID# ___________________________

Date:__________________________

PedsQL™

Pediatric Quality of Life Inventory

Version 4.0

YOUNG CHILD REPORT (ages **5-7**)

Instructions for interviewer:

I am going to ask you some questions about things that might be a problem for some children. I want to know how much of a problem any of these things might be for you.

Show the child the template and point to the responses as you read.

If it is <u>not at all</u> a problem for you, point to the smiling face

If it is <u>sometimes</u> a problem for you, point to the middle face

If it is a problem for you <u>a lot</u>, point to the frowning face

I will read each question. Point to the pictures to show me how much of a problem it is for you. Let's try a practice one first.

	Not at all	Sometimes	A lot
**Is it hard for you to snap your fingers**	☺	😐	☹

Ask the child to demonstrate snapping his or her fingers to determine whether or not the question was answered correctly. Repeat the question if the child demonstrates a response that is different from his or her action.

PedsQL 4.0 - (5-7) Not to be reproduced without permission Copyright © 1998 JW Varni, Ph.D. All rights reserved
01/00
PedsQL-4.0-Core-YC – United States/English – Original version
PedsQL-4.0-Core-YC_AU4.0_eng-USori

Think about how you have been doing for the last few weeks. Please listen carefully to each sentence and tell me how much of a problem this is for you.

After reading the item, gesture to the template. If the child hesitates or does not seem to understand how to answer, read the response options while pointing at the faces.

**PHYSICAL FUNCTIONING *(problems with…)***	**Not at all**	**Some-times**	**A lot**
1. Is it hard for you to walk	0	2	4
2. Is it hard for you to run	0	2	4
3. Is it hard for you to play sports or exercise	0	2	4
4. Is it hard for you to pick up big things	0	2	4
5. Is it hard for you to take a bath or shower	0	2	4
6. Is it hard for you to do chores (like pick up your toys)	0	2	4
7. Do you have hurts or aches (***Where?***___________)	0	2	4
8. Do you ever feel too tired to play	0	2	4

Remember, tell me how much of a problem this has been for you for the last few weeks.

**EMOTIONAL FUNCTIONING *(problems with…)***	**Not at all**	**Some-times**	**A lot**
1. Do you feel scared	0	2	4
2. Do you feel sad	0	2	4
3. Do you feel mad	0	2	4
4. Do you have trouble sleeping	0	2	4
5. Do you worry about what will happen to you	0	2	4

**SOCIAL FUNCTIONING *(problems with…)***	**Not at all**	**Some-times**	**A lot**
1. Is it hard for you to get along with other kids	0	2	4
2. Do other kids say they do not want to play with you	0	2	4
3. Do other kids tease you	0	2	4
4. Can other kids do things that you cannot do	0	2	4
5. Is it hard for you to keep up when you play with other kids	0	2	4

**SCHOOL FUNCTIONING *(problems with…)***	**Not at all**	**Some-times**	**A lot**
1. Is it hard for you to pay attention in school	0	2	4
2. Do you forget things	0	2	4
3. Is it hard to keep up with schoolwork	0	2	4
4. Do you miss school because of not feeling good	0	2	4
5. Do you miss school because you have to go to the doctor’s or hospital	0	2	4

PedsQL 4.0 - (5-7) Not to be reproduced without permission Copyright © 1998 JW Varni, Ph.D. All rights reserved
01/00
PedsQL-4.0-Core-YC – United States/English – Original version
PedsQL-4.0-Core-YC_AU4.0_eng-USori

How much of a problem is this for you?

Not at all	Sometimes	A lot
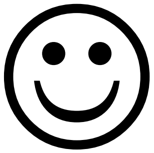	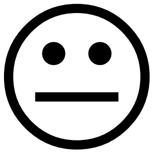	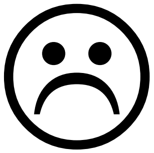

Not to be reproduced without permission
Copyright © 1998 JW Varni, Ph.D. All rights reserved.

ID# ___________________________

Date:___________________________

PedsQL ™

Pediatric Quality of Life Inventory

Version 4.0

PARENT REPORT for **YOUNG CHILDREN** (ages **5-7**)

DIRECTIONS

On the following page is a list of things that might be a problem for **your child**. Please tell us **how much of a problem** each one has been for **your child** during the **past ONE month** by circling:

0 if it is **never** a problem
1 if it is **almost never** a problem
2 if it is **sometimes** a problem
3 if it is **often** a problem
4 if it is **almost always** a problem

There are no right or wrong answers.
If you do not understand a question, please ask for help.

Copyright © 1998 JW Varni, Ph.D. All rights reserved

*In the past **ONE month,** how much of a **problem** has your child had with …*

**PHYSICAL FUNCTIONING *(problems with…)***	**Never**	**Almost Never**	**Some-times**	**Often**	**Almost Always**
1. Walking more than one block	0	1	2	3	4
2. Running	0	1	2	3	4
3. Participating in sports activity or exercise	0	1	2	3	4
4. Lifting something heavy	0	1	2	3	4
5. Taking a bath or shower by him or herself	0	1	2	3	4
6. Doing chores, like picking up his or her toys	0	1	2	3	4
7. Having hurts or aches	0	1	2	3	4
8. Low energy level	0	1	2	3	4

**EMOTIONAL FUNCTIONING *(problems with…)***	**Never**	**Almost Never**	**Some-times**	**Often**	**Almost Always**
1. Feeling afraid or scared	0	1	2	3	4
2. Feeling sad or blue	0	1	2	3	4
3. Feeling angry	0	1	2	3	4
4. Trouble sleeping	0	1	2	3	4
5. Worrying about what will happen to him or her	0	1	2	3	4

**SOCIAL FUNCTIONING *(problems with…)***	**Never**	**Almost Never**	**Some-times**	**Often**	**Almost Always**
1. Getting along with other children	0	1	2	3	4
2. Other kids not wanting to be his or her friend	0	1	2	3	4
3. Getting teased by other children	0	1	2	3	4
4. Not able to do things that other children his or her age can do	0	1	2	3	4
5. Keeping up when playing with other children	0	1	2	3	4

**SCHOOL FUNCTIONING *(problems with…)***	**Never**	**Almost Never**	**Some-times**	**Often**	**Almost Always**
1. Paying attention in class	0	1	2	3	4
2. Forgetting things	0	1	2	3	4
3. Keeping up with school activities	0	1	2	3	4
4. Missing school because of not feeling well	0	1	2	3	4
5. Missing school to go to the doctor or hospital	0	1	2	3	4

 Not to be reproduced without permission Copyright © 1998 JW Varni, Ph.D. All rights reserved

ID# ___________________________

Date:__________________________

PedsQL ™

Pediatric Quality of Life Inventory

Version 4.0

PARENT REPORT for **TODDLERS** (ages **2-4**)

DIRECTIONS

On the following page is a list of things that might be a problem for **your child**. Please tell us **how much of a problem** each one has been for **your child** during the **past ONE month** by circling:

0 if it is **never** a problem
1 if it is **almost never** a problem
2 if it is **sometimes** a problem
3 if it is **often** a problem
4 if it is **almost always** a problem

There are no right or wrong answers.
If you do not understand a question, please ask for help.

Not to be reproduced without permission Copyright © 1998 JW Varni, Ph.D. All rights reserved

*In the past **ONE month,** how much of a **problem** has your child had with …*

PHYSICAL FUNCTIONING *(problems with…)*	Never	Almost Never	Some-times	Often	Almost Always
1. Walking	0	1	2	3	4
2. Running	0	1	2	3	4
3. Participating in active play or exercise	0	1	2	3	4
4. Lifting something heavy	0	1	2	3	4
5. Bathing	0	1	2	3	4
6. Helping to pick up his or her toys	0	1	2	3	4
7. Having hurts or aches	0	1	2	3	4
8. Low energy level	0	1	2	3	4

EMOTIONAL FUNCTIONING *(problems with…)*	Never	Almost Never	Some-times	Often	Almost Always
1. Feeling afraid or scared	0	1	2	3	4
2. Feeling sad or blue	0	1	2	3	4
3. Feeling angry	0	1	2	3	4
4. Trouble sleeping	0	1	2	3	4
5. Worrying	0	1	2	3	4

SOCIAL FUNCTIONING *(problems with…)*	Never	Almost Never	Some-times	Often	Almost Always
1. Playing with other children	0	1	2	3	4
2. Other kids not wanting to play with him or her	0	1	2	3	4
3. Getting teased by other children	0	1	2	3	4
4. Not able to do things that other children his or her age can do	0	1	2	3	4
5. Keeping up when playing with other children	0	1	2	3	4

***Please complete this section if your child attends school or daycare**

SCHOOL FUNCTIONING *(problems with…)*	Never	Almost Never	Some-times	Often	Almost Always
1. Doing the same school activities as peers	0	1	2	3	4
2. Missing school/daycare because of not feeling well	0	1	2	3	4
3. Missing school/daycare to go to the doctor or hospital	0	1	2	3	4

 Not to be reproduced without permission Copyright © 1998 JW Varni, Ph.D. All rights reserved

ID# ____________________________

Date:___________________________

PedsQL™

Adult Quality of Life Inventory

Version 4.0

ADULT REPORT
Acute Version

DIRECTIONS

On the following page is a list of things that might be a problem for you. Please tell us **how much of a problem** each one has been for you during the **past 7 days** by circling:

0 if it is **never** a problem
1 if it is **almost never** a problem
2 if it is **sometimes** a problem
3 if it is **often** a problem
4 if it is **almost always** a problem

There are no right or wrong answers.
If you do not understand a question, please ask for help.

 Copyright © 1998 JW Varni, Ph.D. All rights reserved

*In the past **7 days**, how much of a **problem** has this been for you …*

**ABOUT MY HEALTH AND ACTIVITIES *(problems with…)***	Never	Almost Never	Some-times	Often	Almost Always
1. It is hard for me to walk more than one block	0	1	2	3	4
2. It is hard for me to run	0	1	2	3	4
3. It is hard for me to do sports activity or exercise	0	1	2	3	4
4. It is hard for me to lift something heavy	0	1	2	3	4
5. It is hard for me to take a bath or shower by myself	0	1	2	3	4
6. It is hard for me to do chores around the house	0	1	2	3	4
7. I hurt or ache	0	1	2	3	4
8. I have low energy	0	1	2	3	4

**ABOUT MY FEELINGS *(problems with…)***	Never	Almost Never	Some-times	Often	Almost Always
1. I feel afraid or scared	0	1	2	3	4
2. I feel sad or blue	0	1	2	3	4
3. I feel angry	0	1	2	3	4
4. I have trouble sleeping	0	1	2	3	4
5. I worry about what will happen to me	0	1	2	3	4

**HOW I GET ALONG WITH OTHERS *(problems with…)***	Never	Almost Never	Some-times	Often	Almost Always
1. I have trouble getting along with other adults	0	1	2	3	4
2. Other adults do not want to be my friend	0	1	2	3	4
3. Other adults tease me	0	1	2	3	4
4. I cannot do things that others my age can do	0	1	2	3	4
5. It is hard to keep up with my peers	0	1	2	3	4

**ABOUT MY WORK/STUDIES *(problems with…)***	Never	Almost Never	Some-times	Often	Almost Always
1. It is hard to pay attention at work or school	0	1	2	3	4
2. I forget things	0	1	2	3	4
3. I have trouble keeping up with my work or studies	0	1	2	3	4
4. I miss work or school because of not feeling well	0	1	2	3	4
5. I miss work or school to go to the doctor or hospital	0	1	2	3	4

Not to be reproduced without permission Copyright © 1998 JW Varni, Ph.D. All rights reserved

ID#______________________________

Date:____________________________

PedsQL™

Adult Quality of Life Inventory

Version 4.0

PARENT REPORT for **ADULTS**

Acute Version

DIRECTIONS

On the following page is a list of things that might be a problem for **your child**. Please tell us **how much of a problem** each one has been for **your child** during the **past 7 days** by circling:

0 if it is **never** a problem
1 if it is **almost never** a problem
2 if it is **sometimes** a problem
3 if it is **often** a problem
4 if it is **almost always** a problem

There are no right or wrong answers.
If you do not understand a question, please ask for help.

Copyright © 1998 JW Varni, Ph.D. All rights reserved

*In the past **7 days**, how much of a **problem** has your child had with …*

PHYSICAL FUNCTIONING *(problems with…)*	Never	Almost Never	Some-times	Often	Almost Always
1. Walking more than one block	0	1	2	3	4
2. Running	0	1	2	3	4
3. Participating in sports activity or exercise	0	1	2	3	4
4. Lifting something heavy	0	1	2	3	4
5. Taking a bath or shower by him or herself	0	1	2	3	4
6. Doing chores around the house	0	1	2	3	4
7. Having hurts or aches	0	1	2	3	4
8. Low energy level	0	1	2	3	4

EMOTIONAL FUNCTIONING *(problems with…)*	Never	Almost Never	Some-times	Often	Almost Always
1. Feeling afraid or scared	0	1	2	3	4
2. Feeling sad or blue	0	1	2	3	4
3. Feeling angry	0	1	2	3	4
4. Trouble sleeping	0	1	2	3	4
5. Worrying about what will happen to him or her	0	1	2	3	4

SOCIAL FUNCTIONING *(problems with…)*	Never	Almost Never	Some-times	Often	Almost Always
1. Getting along with other adults	0	1	2	3	4
2. Other adults not wanting to be his or her friend	0	1	2	3	4
3. Getting teased by other adults	0	1	2	3	4
4. Not able to do things that others his or her age can do	0	1	2	3	4
5. Keeping up with other adults	0	1	2	3	4

WORK/STUDIES FUNCTIONING *(problems with…)*	Never	Almost Never	Some-times	Often	Almost Always
1. Paying attention at work or school	0	1	2	3	4
2. Forgetting things	0	1	2	3	4
3. Keeping up with work or studies	0	1	2	3	4
4. Missing work or school because of not feeling well	0	1	2	3	4
5. Missing work or school to go to the doctor or hospital	0	1	2	3	4

Not to be reproduced without permission Copyright © 1998 JW Varni, Ph.D. All rights reserved

ID# ___________________________

Date:___________________________

PedsQL™

Young Adult Quality of Life Inventory

Version 4.0

YOUNG ADULT REPORT (ages **18-25**)
Acute Version

DIRECTIONS

On the following page is a list of things that might be a problem for you. Please tell us **how much of a problem** each one has been for you during the **past 7 days** by circling:

0 if it is **never** a problem
1 if it is **almost never** a problem
2 if it is **sometimes** a problem
3 if it is **often** a problem
4 if it is **almost always** a problem

There are no right or wrong answers.
If you do not understand a question, please ask for help.

 Copyright © 1998 JW Varni, Ph.D. All rights reserved

*In the past **7 days**, how much of a **problem** has this been for you …*

**ABOUT MY HEALTH AND ACTIVITIES *(problems with…)***	**Never**	**Almost Never**	**Some-times**	**Often**	**Almost Always**
1. It is hard for me to walk more than one block	0	1	2	3	4
2. It is hard for me to run	0	1	2	3	4
3. It is hard for me to do sports activity or exercise	0	1	2	3	4
4. It is hard for me to lift something heavy	0	1	2	3	4
5. It is hard for me to take a bath or shower by myself	0	1	2	3	4
6. It is hard for me to do chores around the house	0	1	2	3	4
7. I hurt or ache	0	1	2	3	4
8. I have low energy	0	1	2	3	4

**ABOUT MY FEELINGS *(problems with…)***	**Never**	**Almost Never**	**Some-times**	**Often**	**Almost Always**
1. I feel afraid or scared	0	1	2	3	4
2. I feel sad or blue	0	1	2	3	4
3. I feel angry	0	1	2	3	4
4. I have trouble sleeping	0	1	2	3	4
5. I worry about what will happen to me	0	1	2	3	4

**HOW I GET ALONG WITH OTHERS *(problems with…)***	**Never**	**Almost Never**	**Some-times**	**Often**	**Almost Always**
1. I have trouble getting along with other young adults	0	1	2	3	4
2. Other young adults do not want to be my friend	0	1	2	3	4
3. Other young adults tease me	0	1	2	3	4
4. I cannot do things that others my age can do	0	1	2	3	4
5. It is hard to keep up with my peers	0	1	2	3	4

**ABOUT MY WORK/STUDIES *(problems with…)***	**Never**	**Almost Never**	**Some-times**	**Often**	**Almost Always**
1. It is hard to pay attention at work or school	0	1	2	3	4
2. I forget things	0	1	2	3	4
3. I have trouble keeping up with my work or studies	0	1	2	3	4
4. I miss work or school because of not feeling well	0	1	2	3	4
5. I miss work or school to go to the doctor or hospital	0	1	2	3	4

Not to be reproduced without permission

Copyright © 1998 JW Varni, Ph.D. All rights reserved

ID#___________________________

Date:_________________________

PedsQL ™

Young Adult Quality of Life Inventory

Version 4.0

PARENT REPORT for **YOUNG ADULTS** (ages **18-25**)

Acute Version

DIRECTIONS

On the following page is a list of things that might be a problem for **your child**. Please tell us **how much of a problem** each one has been for **your child** during the **past 7 days** by circling:

0 if it is **never** a problem
1 if it is **almost never** a problem
2 if it is **sometimes** a problem
3 if it is **often** a problem
4 if it is **almost always** a problem

There are no right or wrong answers.
If you do not understand a question, please ask for help.

 Not to be reproduced without permission Copyright © 1998 JW Varni, Ph.D. All rights reserved

*In the past **7 days,** how much of a **problem** has your child had with …*

PHYSICAL FUNCTIONING *(problems with…)*	Never	Almost Never	Some-times	Often	Almost Always
1. Walking more than one block	0	1	2	3	4
2. Running	0	1	2	3	4
3. Participating in sports activity or exercise	0	1	2	3	4
4. Lifting something heavy	0	1	2	3	4
5. Taking a bath or shower by him or herself	0	1	2	3	4
6. Doing chores around the house	0	1	2	3	4
7. Having hurts or aches	0	1	2	3	4
8. Low energy level	0	1	2	3	4

EMOTIONAL FUNCTIONING *(problems with…)*	Never	Almost Never	Some-times	Often	Almost Always
1. Feeling afraid or scared	0	1	2	3	4
2. Feeling sad or blue	0	1	2	3	4
3. Feeling angry	0	1	2	3	4
4. Trouble sleeping	0	1	2	3	4
5. Worrying about what will happen to him or her	0	1	2	3	4

SOCIAL FUNCTIONING *(problems with…)*	Never	Almost Never	Some-times	Often	Almost Always
1. Getting along with other young adults	0	1	2	3	4
2. Other young adults not wanting to be his or her friend	0	1	2	3	4
3. Getting teased by other young adults	0	1	2	3	4
4. Not able to do things that others his or her age can do	0	1	2	3	4
5. Keeping up with other young adults	0	1	2	3	4

WORK/STUDIES FUNCTIONING *(problems with…)*	Never	Almost Never	Some-times	Often	Almost Always
1. Paying attention at work or school	0	1	2	3	4
2. Forgetting things	0	1	2	3	4
3. Keeping up with work or studies	0	1	2	3	4
4. Missing work or school because of not feeling well	0	1	2	3	4
5. Missing work or school to go to the doctor or hospital	0	1	2	3	4

ID# ___________________________

Date:__________________________

PedsQL ™

Pediatric Quality of Life Inventory

Version 4.0

TEEN REPORT (ages **13-18**)
Acute Version

DIRECTIONS

On the following page is a list of things that might be a problem for you. Please tell us **how much of a problem** each one has been for you during the **past 7 days** by circling:

0 if it is **never** a problem
1 if it is **almost never** a problem
2 if it is **sometimes** a problem
3 if it is **often** a problem
4 if it is **almost always** a problem

There are no right or wrong answers.
If you do not understand a question, please ask for help.

Not to be reproduced without permission
Copyright © 1998 JW Varni, PhD.
All rights reserved

*In the past **7 days**, how much of a **problem** has this been for you …*

**ABOUT MY HEALTH AND ACTIVITIES *(problems with…)***	**Never**	**Almost Never**	**Some-times**	**Often**	**Almost Always**
1. It is hard for me to walk more than one block	0	1	2	3	4
2. It is hard for me to run	0	1	2	3	4
3. It is hard for me to do sports activity or exercise	0	1	2	3	4
4. It is hard for me to lift something heavy	0	1	2	3	4
5. It is hard for me to take a bath or shower by myself	0	1	2	3	4
6. It is hard for me to do chores around the house	0	1	2	3	4
7. I hurt or ache	0	1	2	3	4
8. I have low energy	0	1	2	3	4

**ABOUT MY FEELINGS *(problems with…)***	**Never**	**Almost Never**	**Some-times**	**Often**	**Almost Always**
1. I feel afraid or scared	0	1	2	3	4
2. I feel sad or blue	0	1	2	3	4
3. I feel angry	0	1	2	3	4
4. I have trouble sleeping	0	1	2	3	4
5. I worry about what will happen to me	0	1	2	3	4

**HOW I GET ALONG WITH OTHERS *(problems with…)***	**Never**	**Almost Never**	**Some-times**	**Often**	**Almost Always**
1. I have trouble getting along with other teens	0	1	2	3	4
2. Other teens do not want to be my friend	0	1	2	3	4
3. Other teens tease me	0	1	2	3	4
4. I cannot do things that other teens my age can do	0	1	2	3	4
5. It is hard to keep up with my peers	0	1	2	3	4

**ABOUT SCHOOL *(problems with…)***	**Never**	**Almost Never**	**Some-times**	**Often**	**Almost Always**
1. It is hard to pay attention in class	0	1	2	3	4
2. I forget things	0	1	2	3	4
3. I have trouble keeping up with my schoolwork	0	1	2	3	4
4. I miss school because of not feeling well	0	1	2	3	4
5. I miss school to go to the doctor or hospital	0	1	2	3	4

Not to be reproduced without permission
Copyright © 1998 JW Varni, PhD.
All rights reserved

ID#__________________________

Date:________________________

PedsQL™

Pediatric Quality of Life Inventory

Version 4.0

PARENT REPORT for **TEENS** (ages **13-18**)
Acute Version

DIRECTIONS

On the following page is a list of things that might be a problem for **your teen**. Please tell us **how much of a problem** each one has been for **your teen** during the **past 7 days** by circling:

0 if it is **never** a problem
1 if it is **almost never** a problem
2 if it is **sometimes** a problem
3 if it is **often** a problem
4 if it is **almost always** a problem

There are no right or wrong answers.
If you do not understand a question, please ask for help.

Not to be reproduced without permission
Copyright © 1998 JW Varni, PhD.
All rights reserved

*In the past **7 days,** how much of a **problem** has your teen had with …*

**PHYSICAL FUNCTIONING *(problems with…)***	**Never**	**Almost Never**	**Some-times**	**Often**	**Almost Always**
1. Walking more than one block	0	1	2	3	4
2. Running	0	1	2	3	4
3. Participating in sports activity or exercise	0	1	2	3	4
4. Lifting something heavy	0	1	2	3	4
5. Taking a bath or shower by him or herself	0	1	2	3	4
6. Doing chores around the house	0	1	2	3	4
7. Having hurts or aches	0	1	2	3	4
8. Low energy level	0	1	2	3	4

**EMOTIONAL FUNCTIONING *(problems with…)***	**Never**	**Almost Never**	**Some-times**	**Often**	**Almost Always**
1. Feeling afraid or scared	0	1	2	3	4
2. Feeling sad or blue	0	1	2	3	4
3. Feeling angry	0	1	2	3	4
4. Trouble sleeping	0	1	2	3	4
5. Worrying about what will happen to him or her	0	1	2	3	4

**SOCIAL FUNCTIONING *(problems with…)***	**Never**	**Almost Never**	**Some-times**	**Often**	**Almost Always**
1. Getting along with other teens	0	1	2	3	4
2. Other teens not wanting to be his or her friend	0	1	2	3	4
3. Getting teased by other teens	0	1	2	3	4
4. Not able to do things that other teens his or her age can do	0	1	2	3	4
5. Keeping up with other teens	0	1	2	3	4

**SCHOOL FUNCTIONING *(problems with…)***	**Never**	**Almost Never**	**Some-times**	**Often**	**Almost Always**
1. Paying attention in class	0	1	2	3	4
2. Forgetting things	0	1	2	3	4
3. Keeping up with schoolwork	0	1	2	3	4
4. Missing school because of not feeling well	0	1	2	3	4
5. Missing school to go to the doctor or hospital	0	1	2	3	4

Not to be reproduced without permission

Copyright © 1998 JW Varni, PhD.
All rights reserved

ID# ___________________________

Date:___________________________

PedsQL ™

Pediatric Quality of Life Inventory

Version 4.0

CHILD REPORT (ages **8-12**)
Acute Version

DIRECTIONS

On the following page is a list of things that might be a problem for you. Please tell us **how much of a problem** each one has been for you during the **past 7 days** by circling:

0 if it is **never** a problem
1 if it is **almost never** a problem
2 if it is **sometimes** a problem
3 if it is **often** a problem
4 if it is **almost always** a problem

There are no right or wrong answers.
If you do not understand a question, please ask for help.

Not to be reproduced without permission
Copyright © 1998 JW Varni, PhD.
All rights reserved

*In the past **7 days**, how much of a **problem** has this been for you …*

ABOUT MY HEALTH AND ACTIVITIES ***(problems with…)***	**Never**	**Almost Never**	**Some-times**	**Often**	**Almost Always**
1. It is hard for me to walk more than one block	0	1	2	3	4
2. It is hard for me to run	0	1	2	3	4
3. It is hard for me to do sports activity or exercise	0	1	2	3	4
4. It is hard for me to lift something heavy	0	1	2	3	4
5. It is hard for me to take a bath or shower by myself	0	1	2	3	4
6. It is hard for me to do chores around the house	0	1	2	3	4
7. I hurt or ache	0	1	2	3	4
8. I have low energy	0	1	2	3	4

ABOUT MY FEELINGS ***(problems with…)***	**Never**	**Almost Never**	**Some-times**	**Often**	**Almost Always**
1. I feel afraid or scared	0	1	2	3	4
2. I feel sad or blue	0	1	2	3	4
3. I feel angry	0	1	2	3	4
4. I have trouble sleeping	0	1	2	3	4
5. I worry about what will happen to me	0	1	2	3	4

HOW I GET ALONG WITH OTHERS ***(problems with…)***	**Never**	**Almost Never**	**Some-times**	**Often**	**Almost Always**
1. I have trouble getting along with other kids	0	1	2	3	4
2. Other kids do not want to be my friend	0	1	2	3	4
3. Other kids tease me	0	1	2	3	4
4. I cannot do things that other kids my age can do	0	1	2	3	4
5. It is hard to keep up when I play with other kids	0	1	2	3	4

ABOUT SCHOOL ***(problems with…)***	**Never**	**Almost Never**	**Some-times**	**Often**	**Almost Always**
1. It is hard to pay attention in class	0	1	2	3	4
2. I forget things	0	1	2	3	4
3. I have trouble keeping up with my schoolwork	0	1	2	3	4
4. I miss school because of not feeling well	0	1	2	3	4
5. I miss school to go to the doctor or hospital	0	1	2	3	4

Not to be reproduced without permission
Copyright © 1998 JW Varni, PhD.
All rights reserved

ID# ___________________________

Date:__________________________

PedsQL™

Pediatric Quality of Life Inventory

Version 4.0

PARENT REPORT for **CHILDREN** (ages **8-12**)
Acute Version

DIRECTIONS

On the following page is a list of things that might be a problem for **your child**. Please tell us **how much of a problem** each one has been for **your child** during the **past 7 days** by circling:

0 if it is **never** a problem
1 if it is **almost never** a problem
2 if it is **sometimes** a problem
3 if it is **often** a problem
4 if it is **almost always** a problem

There are no right or wrong answers.
If you do not understand a question, please ask for help.

Not to be reproduced without permission

Copyright © 1998 JW Varni, PhD.
All rights reserved

*In the past **7 days,** how much of a **problem** has your child had with …*

**PHYSICAL FUNCTIONING *(problems with…)***	**Never**	**Almost Never**	**Some-times**	**Often**	**Almost Always**
1. Walking more than one block	0	1	2	3	4
2. Running	0	1	2	3	4
3. Participating in sports activity or exercise	0	1	2	3	4
4. Lifting something heavy	0	1	2	3	4
5. Taking a bath or shower by him or herself	0	1	2	3	4
6. Doing chores around the house	0	1	2	3	4
7. Having hurts or aches	0	1	2	3	4
8. Low energy level	0	1	2	3	4

**EMOTIONAL FUNCTIONING *(problems with…)***	**Never**	**Almost Never**	**Some-times**	**Often**	**Almost Always**
1. Feeling afraid or scared	0	1	2	3	4
2. Feeling sad or blue	0	1	2	3	4
3. Feeling angry	0	1	2	3	4
4. Trouble sleeping	0	1	2	3	4
5. Worrying about what will happen to him or her	0	1	2	3	4

**SOCIAL FUNCTIONING *(problems with…)***	**Never**	**Almost Never**	**Some-times**	**Often**	**Almost Always**
1. Getting along with other children	0	1	2	3	4
2. Other kids not wanting to be his or her friend	0	1	2	3	4
3. Getting teased by other children	0	1	2	3	4
4. Not able to do things that other children his or her age can do	0	1	2	3	4
5. Keeping up when playing with other children	0	1	2	3	4

**SCHOOL FUNCTIONING *(problems with…)***	**Never**	**Almost Never**	**Some-times**	**Often**	**Almost Always**
1. Paying attention in class	0	1	2	3	4
2. Forgetting things	0	1	2	3	4
3. Keeping up with schoolwork	0	1	2	3	4
4. Missing school because of not feeling well	0	1	2	3	4
5. Missing school to go to the doctor or hospital	0	1	2	3	4

Not to be reproduced without permission
Copyright © 1998 JW Varni, PhD.
All rights reserved

ID# ___________________________

Date:___________________________

PedsQL™

Pediatric Quality of Life Inventory

Version 4.0

YOUNG CHILD REPORT (ages **5-7**)

Acute Version

Instructions for interviewer:

I am going to ask you some questions about things that might be a problem for some children. I want to know how much of a problem any of these things might be for you.

Show the child the template and point to the responses as you read.

If it is not at all a problem for you, point to the smiling face

If it is sometimes a problem for you, point to the middle face

If it is a problem for you a lot, point to the frowning face

I will read each question. Point to the pictures to show me how much of a problem it is for you. Let's try a practice one first.

	Not at all	Sometimes	A lot
**Is it hard for you to snap your fingers**	☺	😐	☹

Ask the child to demonstrate snapping his or her fingers to determine whether or not the question was answered correctly. Repeat the question if the child demonstrates a response that is different from his or her action.

Not to be reproduced without permission

Copyright © 1998 JW Varni, PhD.
All rights reserved

Think about how you have been doing for the past 7 days. Please listen carefully to each sentence and tell me how much of a problem this is for you.

After reading the item, gesture to the template. If the child hesitates or does not seem to understand how to answer, read the response options while pointing at the faces.

**PHYSICAL FUNCTIONING *(problems with…)***	**Not at all**	**Some-times**	**A lot**
1. Is it hard for you to walk	0	2	4
2. Is it hard for you to run	0	2	4
3. Is it hard for you to play sports or exercise	0	2	4
4. Is it hard for you to pick up big things	0	2	4
5. Is it hard for you to take a bath or shower	0	2	4
6. Is it hard for you to do chores (like pick up your toys)	0	2	4
7. Do you have hurts or aches (***Where?***___________)	0	2	4
8. Do you ever feel too tired to play	0	2	4

Remember, tell me how much of a problem this has been for you for the past 7 days.

**EMOTIONAL FUNCTIONING *(problems with…)***	**Not at all**	**Some-times**	**A lot**
1. Do you feel scared	0	2	4
2. Do you feel sad	0	2	4
3. Do you feel mad	0	2	4
4. Do you have trouble sleeping	0	2	4
5. Do you worry about what will happen to you	0	2	4

**SOCIAL FUNCTIONING *(problems with…)***	**Not at all**	**Some-times**	**A lot**
1. Is it hard for you to get along with other kids	0	2	4
2. Do other kids say they do not want to play with you	0	2	4
3. Do other kids tease you	0	2	4
4. Can other kids do things that you cannot do	0	2	4
5. Is it hard for you to keep up when you play with other kids	0	2	4

**SCHOOL FUNCTIONING *(problems with…)***	**Not at all**	**Some-times**	**A lot**
1. Is it hard for you to pay attention in school	0	2	4
2. Do you forget things	0	2	4
3. Is it hard to keep up with schoolwork	0	2	4
4. Do you miss school because of not feeling good	0	2	4
5. Do you miss school because you have to go to the doctor's or hospital	0	2	4

Not to be reproduced without permission
Copyright © 1998 JW Varni, PhD.
All rights reserved

How much of a problem is this for you?

Not at all	Sometimes	A lot
☺	😐	☹

Not to be reproduced without permission
Copyright © 1998 JW Varni, PhD.
All rights reserved

ID# ___________________________

Date:___________________________

PedsQL™

Pediatric Quality of Life Inventory

Version 4.0

PARENT REPORT for **YOUNG CHILDREN** (ages **5-7**)
Acute Version

DIRECTIONS

On the following page is a list of things that might be a problem for **your child**. Please tell us **how much of a problem** each one has been for **your child** during the **past 7 days** by circling:

0 if it is **never** a problem
1 if it is **almost never** a problem
2 if it is **sometimes** a problem
3 if it is **often** a problem
4 if it is **almost always** a problem

There are no right or wrong answers.
If you do not understand a question, please ask for help.

Not to be reproduced without permission
Copyright © 1998 JW Varni, PhD.
All rights reserved

*In the past **7 days,** how much of a **problem** has your child had with …*

**PHYSICAL FUNCTIONING *(problems with…)***	**Never**	**Almost Never**	**Some-times**	**Often**	**Almost Always**
1. Walking more than one block	0	1	2	3	4
2. Running	0	1	2	3	4
3. Participating in sports activity or exercise	0	1	2	3	4
4. Lifting something heavy	0	1	2	3	4
5. Taking a bath or shower by him or herself	0	1	2	3	4
6. Doing chores, like picking up his or her toys	0	1	2	3	4
7. Having hurts or aches	0	1	2	3	4
8. Low energy level	0	1	2	3	4

**EMOTIONAL FUNCTIONING *(problems with…)***	**Never**	**Almost Never**	**Some-times**	**Often**	**Almost Always**
1. Feeling afraid or scared	0	1	2	3	4
2. Feeling sad or blue	0	1	2	3	4
3. Feeling angry	0	1	2	3	4
4. Trouble sleeping	0	1	2	3	4
5. Worrying about what will happen to him or her	0	1	2	3	4

**SOCIAL FUNCTIONING *(problems with…)***	**Never**	**Almost Never**	**Some-times**	**Often**	**Almost Always**
1. Getting along with other children	0	1	2	3	4
2. Other kids not wanting to be his or her friend	0	1	2	3	4
3. Getting teased by other children	0	1	2	3	4
4. Not able to do things that other children his or her age can do	0	1	2	3	4
5. Keeping up when playing with other children	0	1	2	3	4

**SCHOOL FUNCTIONING *(problems with…)***	**Never**	**Almost Never**	**Some-times**	**Often**	**Almost Always**
1. Paying attention in class	0	1	2	3	4
2. Forgetting things	0	1	2	3	4
3. Keeping up with school activities	0	1	2	3	4
4. Missing school because of not feeling well	0	1	2	3	4
5. Missing school to go to the doctor or hospital	0	1	2	3	4

Not to be reproduced without permission
Copyright © 1998 JW Varni, PhD.
All rights reserved

ID# ____________________________

Date:___________________________

PedsQL ™

Pediatric Quality of Life Inventory

Version 4.0

PARENT REPORT for **TODDLERS** (ages **2-4**)
Acute Version

DIRECTIONS

On the following page is a list of things that might be a problem for **your child**. Please tell us **how much of a problem** each one has been for **your child** during the **past 7 days** by circling:

0 if it is **never** a problem
1 if it is **almost never** a problem
2 if it is **sometimes** a problem
3 if it is **often** a problem
4 if it is **almost always** a problem

There are no right or wrong answers.
If you do not understand a question, please ask for help.

Not to be reproduced without permission

Copyright © 1998 JW Varni, Ph.D.
All rights reserved

*In the past **7 days,** how much of a **problem** has your child had with …*

**PHYSICAL FUNCTIONING *(problems with…)***	**Never**	**Almost Never**	**Some-times**	**Often**	**Almost Always**
1. Walking	0	1	2	3	4
2. Running	0	1	2	3	4
3. Participating in active play or exercise	0	1	2	3	4
4. Lifting something heavy	0	1	2	3	4
5. Bathing	0	1	2	3	4
6. Helping to pick up his or her toys	0	1	2	3	4
7. Having hurts or aches	0	1	2	3	4
8. Low energy level	0	1	2	3	4

**EMOTIONAL FUNCTIONING *(problems with…)***	**Never**	**Almost Never**	**Some-times**	**Often**	**Almost Always**
1. Feeling afraid or scared	0	1	2	3	4
2. Feeling sad or blue	0	1	2	3	4
3. Feeling angry	0	1	2	3	4
4. Trouble sleeping	0	1	2	3	4
5. Worrying	0	1	2	3	4

**SOCIAL FUNCTIONING *(problems with…)***	**Never**	**Almost Never**	**Some-times**	**Often**	**Almost Always**
1. Playing with other children	0	1	2	3	4
2. Other kids not wanting to play with him or her	0	1	2	3	4
3. Getting teased by other children	0	1	2	3	4
4. Not able to do things that other children his or her age can do	0	1	2	3	4
5. Keeping up when playing with other children	0	1	2	3	4

****Please complete this section if your child attends school or daycare***

**SCHOOL FUNCTIONING *(problems with…)***	**Never**	**Almost Never**	**Some-times**	**Often**	**Almost Always**
1. Doing the same school activities as peers	0	1	2	3	4
2. Missing school/daycare because of not feeling well	0	1	2	3	4
3. Missing school/daycare to go to the doctor or hospital	0	1	2	3	4

Not to be reproduced without permission
Copyright © 1998 JW Varni, Ph.D.
All rights reserved
