# Supplementary material for: Morbidity and Complications of Diabetes Mellitus in Children and Adolescents in Ghana: Protocol for a Longitudinal Study
Source: JMIR Res Protoc. 2021 Jan 6;10(1):e21440. doi: 10.2196/21440 (PMC7817364; doi:10.2196/21440)
Supplement: Multimedia Appendix 4 [file resprot_v10i1e21440_app4.pdf]

User agreement

Special Terms

**Mapi Research Trust**, a non-for-profit organisation subject to the terms of the French law of 1st July 1901, registered in Carpentras under number 453 979 346, whose business address is 27 rue de la Villette, 69003 Lyon, France, hereafter referred to as “MRT” and the User, as defined herein, (each referred to singularly as a “Party” and/or collectively as the “Parties”), do hereby agree to the following User Agreement Special and General Terms:

Mapi Research Trust
PROVIDE™
27 rue de la Villette
69003 Lyon
France
Telephone: +33 (0)4 72 13 65 75

**Recitals**

The User acknowledges that it is subject to these Special Terms and to the General Terms of the Agreement, which are included in Appendix 1 to these Special Terms and fully incorporated herein by reference. Under the Agreement, the Questionnaire referenced herein is licensed, not sold, to the User by MRT for use only in accordance with the terms and conditions defined herein. MRT reserves all rights not expressly granted to the User.

The Parties, in these Special Terms, intend to detail the special conditions of their partnership.

The Parties intend that all capitalized terms in the Special Terms have the same definitions as those given in article 1 of the General Terms included in Appendix 1.

In this respect, the Parties have agreed as follows:

## Article 1. Conditions Specific to the User

### Section 1.01 Identification of the User

| | |
|---|---|
| User Name | Vera Adobea Essuman |
| Legal Form | Individual |
| Address | Ophthalmology Unit, Department of Surgery, School of Medicine and Dentistry, College of Health Sciences, University of Ghana. P. O. Box 4236<br>P. O. Box KB 353, Korle-Bu , Accra, Ghana<br>Greater Accra<br>+233 Accra |
| Country | Ghana |
| Email address | vadessuman@yahoo.com |

### Section 1.02 Identification of the Questionnaire



© Mapi Research Trust. The unauthorized modification and use of any portion of this document is prohibited.

| | |
|---|---|
| Title | Pediatric Quality of Life Inventory™ (PedsQL™) |
| Author(s) | Varni James W |
| Owner | Varni James W, PhD |
| Copyright | Copyright © 1998 JW Varni, Ph.D. All rights reserved |
| Original bibliographic references | See Appendix 2 |

## Article 2. Rights to Use

### Section 2.01 Context of the Use of the Questionnaire

The User undertakes to only use the Questionnaire in the context of the Study as defined hereafter.

| | |
|---|---|
| Context of Use | Clinical project or study |
| Title | Morbidity complications of diabetes mellitus in Ghanaian children and adolescents |
| Disease or condition | Diabetes Mellitus |
| Type of research | Epidemiologic/Observational |
| Study/Protocol reference | URF/9/LMG-013/2015-2016 |
| Number of patients expected | Fifty-five (55) |
| Number of submissions to the questionnaire for each patient | One submission per questionnaire per each patient |
| Term of clinical follow-up for each patient | Two years |
| Start | 03/2016 |
| End | 03/2018 |
| Mode of administration | Paper administration |

### Section 2.02 Conditions for Use

The User undertakes to use the Questionnaire in accordance with the conditions for use defined hereafter.

(a) Rights transferred
Acting in the Owner's name, MRT transfers the following limited, non-exclusive rights, to the User (the "Limited Rights")

(i) to use the Questionnaire, only as part of the Study; this right is made up exclusively of the right to communicate it to the Beneficiaries only, free of charge, by any means of communication and by any means of remote distribution known or unknown to date, subject to respecting the conditions for use described hereafter; and

(ii) to reproduce the Questionnaire, only as part of the Study; this right is made up exclusively of the right to physically establish the Questionnaire or to have it physically established, on any paper, electronic, analog or digital medium, and in particular documents, articles, studies, observations, publications, websites whether or not protected by restricted access, CD, DVD, CD-ROM, hard disk, USB flash drive, for the Beneficiaries only and subject to respecting the conditions for use described hereafter; and

(iii) Should the Questionnaire not already have been translated into the language requested, the User is entitled to translate the Questionnaire or have it translated in this language, subject to informing MRT of the same beforehand by the



© Mapi Research Trust. The unauthorized modification and use of any portion of this document is prohibited.

signature of a Translation Agreement indicating the terms of it and to providing a copy of the translation thus obtained as soon as possible to MRT.

The User acknowledges and accepts that it is not entitled to amend, modify, condense, adapt, reorganise the Questionnaire on any medium whatsoever, in any way whatsoever, even minor, without MRT's prior specific written consent.

(b) Specific conditions for the Owner

The Owner has intended to transfer a part of the copyright on the Questionnaire and/or the Documentation to MRT in order to enable MRT to make it available to the User for the purpose of the Study, subject to the User respecting the following conditions:

User shall not modify, abridge, condense, translate, adapt, recast or transform the Questionnaire in any manner or form, including but not limited to any minor or significant change in wordings or organisation in the Questionnaire, without the prior written agreement of the Owner. If permission is granted, any improvements, modifications, or enhancements to the Questionnaire which may be conceived or developed, including translations and modules, shall become the property of the Owner.

The User therefore undertakes to respect these special terms.

(c) Specific conditions for the Questionnaire

- Use in Individual clinical practice or Research study / project

The User undertakes never to duplicate, transfer or publish the Questionnaire without indicating the Copyright Notice.

In the case of use of an electronic version of the Questionnaire in academic studies, the User undertakes to respect the following special obligations:

- In case of use of an IT Company (e-vendor), User shall check with Mapi Research Trust that IT Company has signed the necessary License Agreement with Mapi Research Trust before developing the electronic version of the Questionnaire
- Not modify the questionnaire (items and response scales, including the response scale numbers from 0-4)
- Cite the reference publications
- Insert the Owner's copyright notice on all pages/screens on which the Questionnaire will be presented and insert the Trademark information: PedsQL™, Copyright © 1998 JW Varni, Ph.D. All rights reserved.
- Mention the following information: "PedsQL™ contact information and permission to use: Mapi Research Trust, Lyon, France – Internet: https://eprovide.mapi-trust.org and www.pedsql.org/index.html "
- Submit the screenshots of all the Pages where the Questionnaire appears to Dr James W. Varni before release for approval and to check that the above-mentioned requirements have been respected.

In the case of use of an electronic version of the Questionnaire in commercial studies / projects, the User undertakes to respect the following special obligations:

User shall:

- In case of use of an IT Company (e-vendor), User shall check with Mapi Research Trust that IT Company has signed the necessary License Agreement with Mapi Research Trust before developing the electronic version of the Questionnaire
- Not modify the questionnaire (items and response scales, including the response scale numbers from 0-4)
- Cite the reference publications
- Insert the Owner's copyright notice on all pages/screens on which the Questionnaire will be presented and insert the Trademark information: PedsQL™, Copyright © 1998 JW Varni, Ph.D. All rights reserved.
- Mention the following information: "PedsQL™ contact information and permission to use: Mapi Research Trust, Lyon, France – Internet: https://eprovide.mapi-trust.org and www.pedsql.org/index.html"

- For the first migration of the Questionnaire (generally the original version) into a specific electronic device



© Mapi Research Trust. The unauthorized modification and use of any portion of this document is prohibited.

- Review of screenshots:

After implementation of the Questionnaire into the device, the user/IT Company will generate screen captures (screenshots) of the original questionnaire as displayed in the device. These will be reviewed by Mapi to check that they are consistent with the original paper version in terms of presentation, content and completion except for specific instructions related to the electronic administration. Corrections that may be needed will be reported to the user/IT Company. In this case, screenshots after correction will be generated for another round of review by Mapi until all screenshots are approved.

Dr James W. Varni will review all approved screenshots for a final validation.

- Usability testing:

Usability testing is a methodology which aims to examine whether respondents are able to use a device and associated software as intended. Major issues of concern in usability testing typically include device complexity, navigation and response selection for example.

The objective of this investigation is to ensure that the electronic version of the questionnaire as included in the device meets usability criteria, focusing on functional aspects and respondents' understanding of instructions. Usability testing consists in interviews with patients where patients will complete the electronic version of the Questionnaire on the device and comment on their understanding of the instructions, ease of use and handiness of the device. A Usability testing report presenting results will be produced. If any changes are recommended, these will be implemented by the user/IT Company. If issues raised by respondents are rated as major, the user/IT Company may need to perform additional developments and another round of interviews may be needed.

Dr James W. Varni will review the changes suggested, if any, following the interviews.

The review of screenshots is mandatory. The usability testing is highly recommended by Mapi, however should the User and/or IT Company decide not to perform this step, Mapi Research Trust shall not be held responsible for any consequence and expense associated with this decision which shall remain the User and/or IT Company's sole liability.

The review of screenshots and usability testing, when and if performed, shall be performed exclusively by Mapi and shall be sponsored by the User.

The performance of the review of screenshots and usability testing will result in a certification of the electronic device original version of the Questionnaires by Mapi for future licenses.

- For the migration of other language versions of the Questionnaire on an existing certified specific electronic device

- Update version

After the electronic device original version of the Questionnaire is fully ready, the Questionnaire's language versions developed for paper administration will be updated to reflect the changes in wording of instructions implemented in the electronic device original version of the questionnaire.

Native speakers of the languages will reflect the changes made to the electronic device original version of the Questionnaire and will provide English equivalents of all changes made for Mapi's quality control.

- Review of screenshots:

After implementation of the Questionnaire into the device, the user/IT Company will generate screen captures (screenshots) of



© Mapi Research Trust. The unauthorized modification and use of any portion of this document is prohibited.

the original questionnaire as displayed in the device. These will be reviewed by Mapi to check that they are consistent with the original paper version in terms of presentation, content and completion except for specific instructions related to the electronic administration. Corrections that may be needed will be reported to the user/IT Company. In this case, screenshots after correction will be generated for another round of review by Mapi until all screenshots are approved.

The update of version and review of screenshots are mandatory. These steps shall be performed exclusively by Mapi and shall be sponsored by the User.

The performance of the update of version and review of screenshots will result in a certification of the electronic device language version of the Questionnaires by Mapi for future licenses.

- Use in a publication:

In the case of a publication, article, study or observation on paper or electronic format of the Questionnaire, the User undertakes to respect the following special obligations:

- not to include any full copy of the Questionnaire, but a version with the indication “sample copy, do not use without permission”
- to indicate the name and copyright notice of the Owner (PedsQL™, Copyright © 1998 JW Varni, Ph.D. All rights reserved)
- to include the reference publications of the Questionnaire
- to indicate the details of MRT for any information on the Questionnaire as follows: "PedsQL™ contact information and permission to use: Mapi Research Trust, Lyon, France. – Internet: https://eprovide.mapi-trust.org and www.pedsql.org "
- to provide MRT, as soon as possible, with a copy of any publication regarding the Questionnaire, for information purposes
- to submit the screenshots of all the Pages where the Questionnaire appears to MRT before release to check that the above-mentioned requirements have been respected.

- Use for dissemination or marketing:

In the case of use in a dissemination/marketing context:

- On a website with unrestricted access:

The publication of a copy of the PedsQL™ on a website with unrestricted access is not permitted.

- On a website with restricted access:

In the case of publication on a website with restricted access, the User may include a version of the Questionnaire that may be amended, subject to this version being protected by a sufficiently secure access to only allow the Beneficiaries to access it.

**Article 3. Term**

MRT transfers the Limited Rights to use the Questionnaire as from the date of delivery of the Questionnaire to the User and for the whole period of the Study.

**Article 4. Beneficiaries**

The Parties agree that the User may communicate the Questionnaire in accordance with the conditions defined above to the Beneficiaries involved in the Study only, in relation to the Study defined in section 2.01.



© Mapi Research Trust. The unauthorized modification and use of any portion of this document is prohibited.

**Article 5. Territories and Languages**

MRT transfers the Limited Rights to use the Questionnaire on the following territories and in the languages indicated in the table below:

| Questionnaire | Language |
|---|---|
| PedsQL™ Diabetes module version 3.0 | Afrikaans for South Africa |
| PedsQL™ Diabetes module version 3.0 | Arabic for Algeria |
| PedsQL™ Diabetes module version 3.0 | Arabic for Egypt |
| PedsQL™ Diabetes module version 3.0 | Arabic for Jordan |
| PedsQL™ Diabetes module version 3.0 | Arabic for Kuwait |
| PedsQL™ Diabetes module version 3.0 | Arabic for Lebanon |
| PedsQL™ Diabetes module version 3.0 | Arabic for Morocco |
| PedsQL™ Diabetes module version 3.0 | Arabic for Saudi Arabia |
| PedsQL™ Diabetes module version 3.0 | Bulgarian for Bulgaria |
| PedsQL™ Diabetes module version 3.0 | Czech for Czech Republic |
| PedsQL™ Diabetes module version 3.0 | Danish for Denmark |
| PedsQL™ Diabetes module version 3.0 | Dutch for the Netherlands |
| PedsQL™ Diabetes module version 3.0 | English for India |
| PedsQL™ Diabetes module version 3.0 | English for South Africa |
| PedsQL™ Diabetes module version 3.0 | English for the UK |
| PedsQL™ Diabetes module version 3.0 | English for the USA |
| PedsQL™ Diabetes module version 3.0 | Estonian for Estonia |
| PedsQL™ Diabetes module version 3.0 | Finnish for Finland |
| PedsQL™ Diabetes module version 3.0 | French for Algeria |



© Mapi Research Trust. The unauthorized modification and use of any portion of this document is prohibited.

| | |
|---|---|
| PedsQL™ Diabetes module version 3.0 | French for France |
| PedsQL™ Diabetes module version 3.0 | French for Morocco |
| PedsQL™ Diabetes module version 3.0 | German for Germany |
| PedsQL™ Diabetes module version 3.0 | Hindi for India |
| PedsQL™ Diabetes module version 3.0 | Hungarian for Hungary |
| PedsQL™ Diabetes module version 3.0 | Italian for Italy |
| PedsQL™ Diabetes module version 3.0 | Mandarin for China |
| PedsQL™ Diabetes module version 3.0 | Mandarin for Taiwan |
| PedsQL™ Diabetes module version 3.0 | Polish for Poland |
| PedsQL™ Diabetes module version 3.0 | Portuguese for Portugal |
| PedsQL™ Diabetes module version 3.0 | Romanian for Romania |
| PedsQL™ Diabetes module version 3.0 | Russian for Russia |
| PedsQL™ Diabetes module version 3.0 | Slovenian for Slovenia |
| PedsQL™ Diabetes module version 3.0 | Spanish for Argentina |
| PedsQL™ Diabetes module version 3.0 | Spanish for Chile |
| PedsQL™ Diabetes module version 3.0 | Spanish for Mexico |
| PedsQL™ Diabetes module version 3.0 | Spanish for Spain |
| PedsQL™ Diabetes module version 3.0 | Spanish for the USA |
| PedsQL™ Diabetes module version 3.0 | Swedish for Finland |
| PedsQL™ Diabetes module version 3.0 | Swedish for Sweden |
| PedsQL™ Diabetes module version 3.0 | Xhosa for South Africa |
| PedsQL™ Diabetes module version 3.0 | Zulu for South Africa |
| PedsQL™ Diabetes module version 3.2 | Czech for Czech Republic |



© Mapi Research Trust. The unauthorized modification and use of any portion of this document is prohibited.

| | |
|---|---|
| PedsQL™ Diabetes module version 3.2 | English for the USA |
| PedsQL™ Diabetes module version 3.2 | German for Germany |
| PedsQL™ Diabetes module version 3.2 | Gujarati for India |
| PedsQL™ Diabetes module version 3.2 | Hindi for India |
| PedsQL™ Diabetes module version 3.2 | Marathi for India |
| PedsQL™ Diabetes module version 3.2 | Romanian for Romania |
| PedsQL™ Diabetes module version 3.2 | Spanish for Mexico |
| PedsQL™ Diabetes module version 3.2 | Spanish for Spain |
| PedsQL™ Diabetes module version 3.2 | Spanish for the USA |
| PedsQL™ Diabetes module version 3.2 | Telugu for India |
| PedsQL™ Family Impact module | Arabic for Israel |
| PedsQL™ Family Impact module | Bangla for Bangladesh |
| PedsQL™ Family Impact module | Bulgarian for Bulgaria |
| PedsQL™ Family Impact module | Croatian for Croatia |
| PedsQL™ Family Impact module | Czech for Czech Republic |
| PedsQL™ Family Impact module | English for Australia |
| PedsQL™ Family Impact module | English for Canada |
| PedsQL™ Family Impact module | English for New Zealand |
| PedsQL™ Family Impact module | English for Singapore |
| PedsQL™ Family Impact module | English for the UK |
| PedsQL™ Family Impact module | English for the USA |
| PedsQL™ Family Impact module | Estonian for Estonia |
| PedsQL™ Family Impact module | French for Canada |



© Mapi Research Trust. The unauthorized modification and use of any portion of this document is prohibited.

| | |
|---|---|
| PedsQL™ Family Impact module | French for France |
| PedsQL™ Family Impact module | Georgian for Georgia |
| PedsQL™ Family Impact module | German for Germany |
| PedsQL™ Family Impact module | Greek for Greece |
| PedsQL™ Family Impact module | Hebrew for Israel |
| PedsQL™ Family Impact module | Hungarian for Hungary |
| PedsQL™ Family Impact module | Italian for Italy |
| PedsQL™ Family Impact module | Japanese for Japan |
| PedsQL™ Family Impact module | Korean for Korea |
| PedsQL™ Family Impact module | Latvian for Latvia |
| PedsQL™ Family Impact module | Lithuanian for Lithuania |
| PedsQL™ Family Impact module | Malay for Singapore |
| PedsQL™ Family Impact module | Mandarin for China |
| PedsQL™ Family Impact module | Mandarin for Singapore |
| PedsQL™ Family Impact module | Mandarin for Taiwan |
| PedsQL™ Family Impact module | Polish for Poland |
| PedsQL™ Family Impact module | Portuguese for Brazil |
| PedsQL™ Family Impact module | Romanian for Romania |
| PedsQL™ Family Impact module | Russian for Belarus |
| PedsQL™ Family Impact module | Russian for Israel |
| PedsQL™ Family Impact module | Russian for Russia |
| PedsQL™ Family Impact module | Russian for Ukraine |
| PedsQL™ Family Impact module | Serbian for Bosnia and Herzegovina |



© Mapi Research Trust. The unauthorized modification and use of any portion of this document is prohibited.

| | |
|---|---|
| PedsQL™ Family Impact module | Serbian for Montenegro |
| PedsQL™ Family Impact module | Serbian for Serbia |
| PedsQL™ Family Impact module | Slovenian for Slovenia |
| PedsQL™ Family Impact module | Spanish for Argentina |
| PedsQL™ Family Impact module | Spanish for Chile |
| PedsQL™ Family Impact module | Spanish for Colombia |
| PedsQL™ Family Impact module | Spanish for Mexico |
| PedsQL™ Family Impact module | Spanish for Spain |
| PedsQL™ Family Impact module | Spanish for the USA |
| PedsQL™ Family Impact module | Swedish for Sweden |
| PedsQL™ Family Impact module | Turkish for Turkey |
| PedsQL™ Family Impact module | Twi for Ghana |
| PedsQL™ Family Impact module | Ukrainian for Ukraine |
| PedsQL™ Family Information Form | English for the USA |
| PedsQL™ Family Information Form | Estonian for Estonia |
| PedsQL™ Family Information Form | Mandarin for China |
| PedsQL™ Family Information Form | Russian for Russia |
| PedsQL™ Family Information Form | Spanish for the USA |
| PedsQL™ Generic Core Scales | Afrikaans for South Africa |
| PedsQL™ Generic Core Scales | Arabic for Israel |
| PedsQL™ Generic Core Scales | Arabic for Jordan |
| PedsQL™ Generic Core Scales | Arabic for Kuwait |
| PedsQL™ Generic Core Scales | Arabic for Lebanon |



© Mapi Research Trust. The unauthorized modification and use of any portion of this document is prohibited.

| | |
|---|---|
| PedsQL™ Generic Core Scales | Arabic for Palestine |
| PedsQL™ Generic Core Scales | Arabic for Saudi Arabia |
| PedsQL™ Generic Core Scales | Arabic for Tunisia |
| PedsQL™ Generic Core Scales | Arabic for the United Arab Emirates |
| PedsQL™ Generic Core Scales | Belarusian for Belarus |
| PedsQL™ Generic Core Scales | Bengali for Bangladesh |
| PedsQL™ Generic Core Scales | Bengali for India |
| PedsQL™ Generic Core Scales | Bosnian for Bosnia and Herzegovina |
| PedsQL™ Generic Core Scales | Bulgarian for Bulgaria |
| PedsQL™ Generic Core Scales | Cantonese for Hong Kong |
| PedsQL™ Generic Core Scales | Catalan for Spain |
| PedsQL™ Generic Core Scales | Croatian for Croatia |
| PedsQL™ Generic Core Scales | Cyrillic for Serbia |
| PedsQL™ Generic Core Scales | Czech for Czech Republic |
| PedsQL™ Generic Core Scales | Danish for Denmark |
| PedsQL™ Generic Core Scales | Dutch for Belgium (Flemish) |
| PedsQL™ Generic Core Scales | Dutch for the Netherlands |
| PedsQL™ Generic Core Scales | English for Australia |
| PedsQL™ Generic Core Scales | English for Canada |
| PedsQL™ Generic Core Scales | English for India |
| PedsQL™ Generic Core Scales | English for Israel |
| PedsQL™ Generic Core Scales | English for New Zealand |
| PedsQL™ Generic Core Scales | English for Singapore |



© Mapi Research Trust. The unauthorized modification and use of any portion of this document is prohibited.

| | |
|---|---|
| PedsQL™ Generic Core Scales | English for South Africa |
| PedsQL™ Generic Core Scales | English for the UK |
| PedsQL™ Generic Core Scales | English for the USA |
| PedsQL™ Generic Core Scales | Estonian for Estonia |
| PedsQL™ Generic Core Scales | Farsi for Iran |
| PedsQL™ Generic Core Scales | Finnish for Finland |
| PedsQL™ Generic Core Scales | French for Belgium |
| PedsQL™ Generic Core Scales | French for Canada |
| PedsQL™ Generic Core Scales | French for France |
| PedsQL™ Generic Core Scales | French for Morocco |
| PedsQL™ Generic Core Scales | French for Switzerland |
| PedsQL™ Generic Core Scales | French for Tunisia |
| PedsQL™ Generic Core Scales | Galician for Spain |
| PedsQL™ Generic Core Scales | Georgian for Georgia |
| PedsQL™ Generic Core Scales | German for Austria |
| PedsQL™ Generic Core Scales | German for Belgium |
| PedsQL™ Generic Core Scales | German for Germany |
| PedsQL™ Generic Core Scales | German for Switzerland |
| PedsQL™ Generic Core Scales | Greek for Greece |
| PedsQL™ Generic Core Scales | Gujarati for India |
| PedsQL™ Generic Core Scales | Hebrew for Israel |
| PedsQL™ Generic Core Scales | Hindi for India |
| PedsQL™ Generic Core Scales | Hungarian for Hungary |



© Mapi Research Trust. The unauthorized modification and use of any portion of this document is prohibited.

| | |
|---|---|
| PedsQL™ Generic Core Scales | Indonesian for Indonesia |
| PedsQL™ Generic Core Scales | Italian for Italy |
| PedsQL™ Generic Core Scales | Italian for Switzerland |
| PedsQL™ Generic Core Scales | Japanese for Japan |
| PedsQL™ Generic Core Scales | Konkani for India |
| PedsQL™ Generic Core Scales | Korean for Korea |
| PedsQL™ Generic Core Scales | Latvian for Latvia |
| PedsQL™ Generic Core Scales | Lithuanian for Lithuania |
| PedsQL™ Generic Core Scales | Malay for Malaysia |
| PedsQL™ Generic Core Scales | Malay for Singapore |
| PedsQL™ Generic Core Scales | Mandarin for China |
| PedsQL™ Generic Core Scales | Mandarin for Malaysia |
| PedsQL™ Generic Core Scales | Mandarin for Singapore |
| PedsQL™ Generic Core Scales | Mandarin for Taiwan |
| PedsQL™ Generic Core Scales | Marathi for India |
| PedsQL™ Generic Core Scales | Norwegian for Denmark |
| PedsQL™ Generic Core Scales | Norwegian for Norway |
| PedsQL™ Generic Core Scales | Polish for Poland |
| PedsQL™ Generic Core Scales | Portuguese for Brazil |
| PedsQL™ Generic Core Scales | Portuguese for Portugal |
| PedsQL™ Generic Core Scales | Romanian for Moldavia |
| PedsQL™ Generic Core Scales | Romanian for Romania |
| PedsQL™ Generic Core Scales | Russian for Belarus |



© Mapi Research Trust. The unauthorized modification and use of any portion of this document is prohibited.

| | |
|---|---|
| PedsQL™ Generic Core Scales | Russian for Estonia |
| PedsQL™ Generic Core Scales | Russian for Georgia |
| PedsQL™ Generic Core Scales | Russian for Israel |
| PedsQL™ Generic Core Scales | Russian for Latvia |
| PedsQL™ Generic Core Scales | Russian for Lithuania |
| PedsQL™ Generic Core Scales | Russian for Russia |
| PedsQL™ Generic Core Scales | Russian for Ukraine |
| PedsQL™ Generic Core Scales | Serbian for Bosnia and Herzegovina |
| PedsQL™ Generic Core Scales | Serbian for Montenegro |
| PedsQL™ Generic Core Scales | Serbian for Serbia |
| PedsQL™ Generic Core Scales | Sesotho for South Africa |
| PedsQL™ Generic Core Scales | Slovak for Slovakia |
| PedsQL™ Generic Core Scales | Slovenian for Slovenia |
| PedsQL™ Generic Core Scales | Spanish for Argentina |
| PedsQL™ Generic Core Scales | Spanish for Chile |
| PedsQL™ Generic Core Scales | Spanish for Colombia |
| PedsQL™ Generic Core Scales | Spanish for Mexico |
| PedsQL™ Generic Core Scales | Spanish for Peru |
| PedsQL™ Generic Core Scales | Spanish for Puerto Rico |
| PedsQL™ Generic Core Scales | Spanish for Spain |
| PedsQL™ Generic Core Scales | Spanish for Uruguay |
| PedsQL™ Generic Core Scales | Spanish for Venezuela |
| PedsQL™ Generic Core Scales | Spanish for the USA |



© Mapi Research Trust. The unauthorized modification and use of any portion of this document is prohibited.

| | |
|---|---|
| PedsQL™ Generic Core Scales | Swedish for Denmark |
| PedsQL™ Generic Core Scales | Swedish for Finland |
| PedsQL™ Generic Core Scales | Swedish for Sweden |
| PedsQL™ Generic Core Scales | Tagalog for the Philippines |
| PedsQL™ Generic Core Scales | Tamil for India |
| PedsQL™ Generic Core Scales | Telugu for India |
| PedsQL™ Generic Core Scales | Thai for Thailand |
| PedsQL™ Generic Core Scales | Turkish for Turkey |
| PedsQL™ Generic Core Scales | Ukrainian for Ukraine |
| PedsQL™ Generic Core Scales | Urdu for Pakistan |
| PedsQL™ Generic Core Scales | Xhosa for South Africa |
| PedsQL™ Generic Core Scales | Zulu for South Africa |
| PedsQL™ Short Form 15 Generic Core Scales | Bulgarian for Bulgaria |
| PedsQL™ Short Form 15 Generic Core Scales | Dutch for Belgium (Flemish) |
| PedsQL™ Short Form 15 Generic Core Scales | Dutch for the Netherlands |
| PedsQL™ Short Form 15 Generic Core Scales | English for Australia |
| PedsQL™ Short Form 15 Generic Core Scales | English for Canada |
| PedsQL™ Short Form 15 Generic Core Scales | English for India |
| PedsQL™ Short Form 15 Generic Core Scales | English for New Zealand |
| PedsQL™ Short Form 15 Generic Core Scales | English for the UK |
| PedsQL™ Short Form 15 Generic Core Scales | English for the USA |
| PedsQL™ Short Form 15 Generic Core Scales | Estonian for Estonia |
| PedsQL™ Short Form 15 Generic Core Scales | French for Belgium |



© Mapi Research Trust. The unauthorized modification and use of any portion of this document is prohibited.

| | |
|---|---|
| PedsQL™ Short Form 15 Generic Core Scales | German for Germany |
| PedsQL™ Short Form 15 Generic Core Scales | Greek for Greece |
| PedsQL™ Short Form 15 Generic Core Scales | Hindi for India |
| PedsQL™ Short Form 15 Generic Core Scales | Indonesian for Indonesia |
| PedsQL™ Short Form 15 Generic Core Scales | Italian for Italy |
| PedsQL™ Short Form 15 Generic Core Scales | Romanian for Romania |
| PedsQL™ Short Form 15 Generic Core Scales | Russian for Russia |
| PedsQL™ Short Form 15 Generic Core Scales | Spanish for the USA |
| PedsQL™ Short Form 15 Generic Core Scales | Swahili for Kenya |
| PedsQL™ Short Form 15 Generic Core Scales | Vietnamese for the USA |

**Article 6. Price and Payment Terms**

The User undertakes in relation to MRT to pay the price owed in return for the availability of the Questionnaire, according to the prices set out below, depending on the languages requested and the costs of using the Questionnaire, in accordance with the terms and conditions described in section 6.02 of the General Terms included in Appendix 1.

Access to the Questionnaire in non-funded academic research and individual clinical practice is free of charge.

*Agreed and acknowledged by*

Vera Adobea Essuman

14-Mar-2017

Appendix 1 to the Special Terms: User Agreement General Terms

User has read and accepted the Mapi's General Terms of the Agreement, which are available on MRT's website: https://eprovide.mapi-trust.org/user-agreement-general-terms



© Mapi Research Trust. The unauthorized modification and use of any portion of this document is prohibited.

## Appendix 2 to the Special Terms: References

Generic Core Scales:


- Varni JW, et al. The PedsQL™: Measurement Model for the Pediatric Quality of Life Inventory.Medical Care,1999; 37(2):126-139

- Varni, J.W., et al. The PedsQL™ 4.0: Reliability and validity of the Pediatric Quality of Life Inventory™ Version 4.0 Generic Core Scales in healthy and patient populations. Medical Care,2001; 39(8): 800-812.

- Varni, J.W., et al. (2002). The PedsQL™ 4.0 Generic Core Scales: Sensitivity, responsiveness, and impact on clinical decision-making.Journal of Behavioral Medicine, 25, 175-193.

- Varni, J.W., et al. (2003). The PedsQL™ 4.0 as a pediatric population health measure: Feasibility, reliability, and validity.Ambulatory Pediatrics, 3, 329-341.

- Chan, K.S., Mangione-Smith, R., Burwinkle, T.M., Rosen, M., & Varni, J.W. (2005). The PedsQL™: Reliability and validity of the Short-Form Generic Core Scales and Asthma Module.Medical Care, 43, 256-265.

- Varni, J.W., & Limbers, C.A. (2009). The PedsQL™ 4.0 Generic Core Scales Young Adult Version: Feasibility, reliability and validity in a university student population.Journal of Health Psychology, 14, 611-622.


Asthma Module:


- Varni, J.W., Burwinkle, T.M., Rapoff, M.A., Kamps, J.L., & Olson, N. The PedsQL™ in pediatric asthma: Reliability and validity of the Pediatric Quality of Life Inventory™ Generic Core Scales and Asthma Module. Journal of Behavioral Medicine, 2004; 27:297-318.

- Chan, K.S., Mangione-Smith, R., Burwinkle, T.M, Rosen, M., & Varni, J.W. (2005). The PedsQL™: Reliability and validity of the Short-Form Generic Core Scales and Asthma Module. Medical Care, 43, 256-265.


Brain Tumor Module:


- Palmer, S.N., Meeske, K.A., Katz, E.R., Burwinke, T.M., & Varni, J.W. (2007). The PedsQL™ Brain Tumor Module: Initial reliability and validity. Pediatric Blood and Cancer, 49, 287-293.


Cancer Module:


- Varni, J.W., Burwinkle, T.M., Katz, E.R., Meeske, K., & Dickinson, P. The PedsQL™ in pediatric cancer: Reliability and validity of the Pediatric Quality of Life Inventory™ Generic Core Scales, Multidimensional Fatigue Scale, and Cancer Module.Cancer, 2002; 94: 2090-2106.





© Mapi Research Trust. The unauthorized modification and use of any portion of this document is prohibited.

- Robert RS, Paxton RJ, Palla SL, Yang G, Askins MA, Joy SE, Ater JL. Feasibility, reliability, and validity of the pediatric quality of life inventory™ generic core scales, cancer module, and multidimensional fatigue scale in long-term adult survivors of pediatric cancer.Pediatric Blood & Cancer2012;59:703–707.

Cerebral Palsy Module:

- Varni JW, Burwinkle TM, Berrin SJ, Sherman SA, Artavia K, Malcarne VL, Chambers HG (2006). The PedsQL™ in Pediatric Cerebral Palsy: Reliability, Validity, and Sensitivity of the Generic Core Scales and Cerebral Palsy Module.Developmental Medicine and Child Neurology, 48: 442-449.

Cardiac Module:

- Uzark, K., Jones, K., Burwinkle, T.M., & Varni, J.W. The Pediatric Quality of Life Inventory™ in children with heart disease.Progress in Pediatric Cardiology, 2003; 18:141-148.

- Uzark, K., Jones, K., Slusher, J., Limbers, C.A., Burwinkle, T.M., & Varni, J.W. (2008). Quality of life in children with heart disease as perceived by children and parents. Pediatrics, 121, e1060-e1067.

Cognitive Functioning Scale:

- McCarthy, M.L., MacKenzie, E.J., Durbin, D.R., Aitken, M.E., Jaffe, K.M., Paidas, C.N. et al. (2005). The Pediatric Quality of Life Inventory: An evaluation of its reliability and validity for children with traumatic brain injury.Archives of Physical Medicine and Rehabilitation, 86, 1901-1909.

- Varni, J.W., Burwinkle, T.M., Katz, E.R., Meeske, K., & Dickinson, P. (2002). The PedsQL™ in pediatric cancer: Reliability and validity of the Pediatric Quality of Life Inventory Generic Core Scales, Multidimensional Fatigue Scale, and Cancer Module.Cancer, 94, 2090-2106.

- Varni, J.W., Limbers, C.A., Sorensen, L.G., Neighbors, K., Martz, K., Bucuvalas, J.C., & Alonso, E.M. (2011). PedsQL™ Cognitive Functioning Scale in pediatric liver transplant recipients: Feasibility, reliability and validity.Quality of Life Research, 20, 913–921.

Diabetes Module:

- Varni, J.W., Curtis, B.H., Abetz, L.N., Lasch, K.E., Piault, E.C., & Zeytoonjian, A.A. (2013). Content validity of the PedsQL™ 3.2 Diabetes Module in newly diagnosed patients with Type 1 Diabetes Mellitus ages 8-45.Quality of Life Research. 22, 2169–2181.

- Varni, J.W., Burwinkle, T.M., Jacobs, J.R., Gottschalk, M., Kaufman, F., & Jones, K.L. The PedsQL™ in Type 1 and Type 2 diabetes: Reliability and validity of the Pediatric Quality of Life Inventory™ Generic Core Scales and Type 1 Diabetes Module.Diabetes Care, 2003; 26: 631-637.

- Nansel, T.R., Weisberg-Benchell, J., Wysocki, T., Laffel, L. & Anderson, B. (2008). Quality of life in



© Mapi Research Trust. The unauthorized modification and use of any portion of this document is prohibited.

children with Type 1 diabetes: A comparison of general and disease-specific measures and support for a unitary diabetes quality of life construct. Diabetic Medicine, 25, 1316-1323.

- Naughton, M.J., Ruggiero, A.M., Lawrence, J.M., Imperatore, G., Klingensmith, G.J. Waitzfelder, B., McKeown, R.E., Standiford, D.A., Liese, A.D., & Loots, B. (2008). Health-related quality of life of children and adolescents with type 1 or type 2 diabetes mellitus: SEARCH for Diabetes In Youth Study.Archives of Pediatrics and Adolescent Medicine, 162, 649-657.

- Hilliard, M.E., Lawrence, J.M., Modi, A.C., Anderson, A., Crume, T., Dolan, L.M., Merchant, A.T., Yi-Frazier, J.P., & Hood, K.K. (2013). Identification of minimal clinically important difference scores of the Pediatric Quality of Life Inventory in children, adolescents, and young adults with Type 1 and Type 2 diabetes.Diabetes Care, 36, 1891–1897.

Duchenne Muscular Dystrophy Module:

- Uzark, K., King, E., Cripe, L., Spicer, R., Sage, J., Kinnett, K., Wong, B., Pratt, J., & Varni, J.W. (2012). Health-related quality of life in children and adolescents with Duchenne Muscular Dystrophy.Pediatrics, 130, e1559-e1566.

-

End Stage Renal Disease Module:

- Goldstein, S.L., Graham, N., Warady, B.A., Seikaly, M., McDonald, R., Burwinkle, T.M., Limbers, C.A., & Varni, J.W. (2008). Measuring health-related quality of life in children with ESRD: Performance of the Generic and ESRD-Specific Instrument of the Pediatric Quality of Life Inventory™ (PedsQL™). American Journal of Kidney Diseases, 51, 285-297.

Eosinophilic Esophagitis:

- Franciosi, J.P., Hommel, K.A., Bendo, C.B., King, E.C., Collins, M.H., Eby, M.D., Marsolo, K., Abonia, J.P., von Tiehl, K.F., Putnam, P.E., Greenler, A.J., Greenberg, A.B., Bryson, R.A., Davis, C.M., Olive, A.P., Gupta, S.K., Erwin, E.A., Klinnert, M.D., Spergel, J.M., Denham, J.M., Furuta, G.T., Rothenberg, M.E., & Varni, J.W. (2013). PedsQL™ Eosinophilic Esophagitis Module: Feasibility, reliability and validity.Journal of Pediatric Gastroenterology & Nutrition, 57, 57-66.

- Franciosi, J.P., Hommel, K.A., Greenberg, A.B., Debrosse, C.W., Greenler, A.J., Abonia, J.P., Rothenberg, M.E., & Varni, J.W. (2012). Development of the Pediatric Quality of Life Inventory™ Eosinophilic Esophagitis Module items: Qualitative methods.BMC Gastroenterology, 12:135, 1-8.

- Franciosi J.P., Hommel, K.A., Debrosse, C.W., Greenberg, A.B., Greenler, A.J., Abonia, J.P., Rothenberg, M.E., & Varni, J.W. (2012). Quality of life in paediatric eosinophilic oesophagitis: What is important to patients?Child: Care, Health and Development, 38, 477–483.

Family impact Module:



© Mapi Research Trust. The unauthorized modification and use of any portion of this document is prohibited.

- Varni, J.W., Sherman, S.A., Burwinkle, T.M., Dickinson, P.E., & Dixon, P. (2004). The PedsQL™ Family Impact Module: Preliminary reliability and validity. Health and Quality of Life Outcomes; 2 (55), 1-6.

- Medrano, G.R., Berlin, K.S., & Davies, W.H. (2013). Utility of the PedsQL™ Family Impact Module: Assessing the psychometric properties in a community sample.Quality of Life Research. 22, 2899-2907.

- Jiang, X., Sun, L., Wang, B., Yang, X., Shang, L., & Zhang, Y. (2013). Health-related quality of life among children with recurrent respiratory tract infections in Xi'an, China.PLoS One, 8(2): e56945.

- Mano, K.E., Khan, K.A., Ladwig, R.J., & Weisman, S.J. (2011). The impact of pediatric chronic pain on parents' health-related quality of life and family functioning: Reliability and validity of the PedsQL 4.0 Family Impact Module.Journal of Pediatric Psychology, 36, 517-527.

Gastrointestinal Symptoms Module:

- Varni, J.W., Bendo, C.B., Denham, J., Shulman, R.J., Self, M.M., Neigut, D.A., Nurko S., Patel, A.S, Franciosi, J.P., Saps, M., Verga, B., Smith, A., Yeckes, A., Heinz, N., Langseder, A., Saeed, S., Zacur, G.M., & Pohl, J.F. (in press). PedsQL™ Gastrointestinal Symptoms Module: Feasibility, reliability, and validity.Journal of Pediatric Gastroenterology & Nutrition.

- Varni, J.W., Bendo, C.B., Denham, J., Shulman, R.J., Self, M.M., Neigut, D.A., Nurko, S., Patel, A.S, Franciosi, J.P., Saps, M., Yeckes, A., Langseder, A., Saeed, S., & Pohl, J.F. (in press). PedsQL™ Gastrointestinal Symptoms Scales and Gastrointestinal Worry Scales in pediatric patients with functional and organic gastrointestinal diseases in comparison to healthy controls.Quality of Life Research.

- Varni, J.W., Kay, M.T., Limbers, C.A., Franciosi, J.P., & Pohl, J.F. (2012). PedsQL™ Gastrointestinal Symptoms Module item development: Qualitative methods.Journal of Pediatric Gastroenterology & Nutrition, 54, 664-671.

Gastrointestinal Symptoms Scales:

- Varni, J.W., Bendo, C.B., Denham, J., Shulman, R.J., Self, M.M., Neigut, D.A., Nurko S., Patel, A.S, Franciosi, J.P., Saps, M., Verga, B., Smith, A., Yeckes, A., Heinz, N., Langseder, A., Saeed, S., Zacur, G.M., & Pohl, J.F. (2014). PedsQL™ Gastrointestinal Symptoms Module: Feasibility, reliability, and validity. Journal of Pediatric Gastroenterology & Nutrition,59, 347–355.

- Varni, J.W., Bendo, C.B., Denham, J., Shulman, R.J., Self, M.M., Neigut, D.A., Nurko, S., Patel, A.S, Franciosi, J.P., Saps, M., Yeckes, A., Langseder, A., Saeed, S., & Pohl, J.F. (in press). PedsQL™ Gastrointestinal Symptoms Scales and Gastrointestinal Worry Scales in pediatric patients with functional and organic gastrointestinal diseases in comparison to healthy controls.Quality of Life Research.

- Varni, J.W., Kay, M.T., Limbers, C.A., Franciosi, J.P., & Pohl, J.F. (2012). PedsQL™ Gastrointestinal Symptoms Module item development: Qualitative methods.Journal of Pediatric Gastroenterology & Nutrition, 54, 664-671.



© Mapi Research Trust. The unauthorized modification and use of any portion of this document is prohibited.

General Well-Being Scale:


- Varni, J.W., Seid, M., & Kurtin, P.S. (1999). Pediatric health-related quality of life measurement technology: A guide for health care decision makes. Journal of Clinical Outcomes Management, 6, 33-40.

- Hallstrand, T.S., Curtis, J.R., Aitken, M.L., & Sullivan, S.D. (2003). Quality of life in adolescents with mild asthma. Pediatric Pulmonology, 36, 536-543.


Healthcare Satisfaction Generic Module:


- Varni, J.W., Burwinkle, T.M., Dickinson, P., Sherman, S.A., Dixon, P., Ervice, J.A., Leyden, P.A. & Sadler, B.L. (2004). Evaluation of the built environment at a Children’s Convalescent Hospital: Development of the Pediatric Quality of Life Inventory™ Parent and Staff Satisfaction Measures for pediatric health care facilities. Journal of Developmental and Behavioral Pediatrics, 2004; 25:10-25.

- Li, J., Yuan, L., Wu, Y., Luan, Y., & Hao, Y. (2013). The Chinese version of the Pediatric Quality of Life Inventory™ (PedsQL™) healthcare satisfaction generic module (version 3.0): Psychometric evaluation. Health and Quality of Life Outcomes, 11(1):113.

- de Souza, F.M., Molina, J., Terreri, M.T., Hilário, M.O., & Len, CA. (2012). Reliability of the Pediatric Quality of Life Inventory - Healthcare Satisfaction Generic Module 3.0 version for the assessment of the quality of care of children with chronic diseases. Journal of Pediatrics (Rio J), 88, 54-60.


Health Care Satisfaction Module specific for Hematology/Oncology:


- Varni, J.W., Quiggins, D.J.L., & Ayala, G.X. (2000). Development of the Pediatric Hematology/Oncology Parent Satisfaction survey. Children's Health Care, 29, 243-255.


Infant Scales:


- Varni, J.W., Limbers, C.A., Neighbors, K., Schulz, K., Lieu, J.E.C., Heffer, R.W., Tuzinkiewicz, K., Mangione-Smith, R., Zimmerman, J.J., & Alonso, E.M. (2011). The PedsQL™ Infant Scales: Feasibility, internal consistency reliability and validity in healthy and ill infants.Quality of Life Research, 20, 45-55.

- Grindler, D.J., Blank, S.J., Schulz, K.A., Witsell, D.L., & Lieu, J.E. (2014). Impact of otitis media severity on children's quality of life.Otolaryngology-Head and Neck Surgery, 151, 333–340.

- Bell, N., Kruse, S., Simons, R.K., & Brussoni, M. (2014). A spatial analysis of functional outcomes and quality of life outcomes after pediatric injury.Injury Epidemiology, 1:16, 1-10.


Multidimensional Fatigue Scale:



© Mapi Research Trust. The unauthorized modification and use of any portion of this document is prohibited.

- Varni, J.W., Burwinkle, T.M., Katz, E.R., Meeske, K., & Dickinson, P. (2002). The PedsQL™ in pediatric cancer: Reliability and validity of the Pediatric Quality of Life Inventory™ Generic Core Scales, Multidimensional Fatigue Scale, and Cancer Module.Cancer, 94, 2090-2106.

- Varni, J. W., Beaujean, A., & Limbers, C. A. (2013). Factorial invariance of pediatric patient self-reported fatigue across age and gender: A multigroup confirmatory factor analysis approach utilizing the PedsQL™ Multidimensional Fatigue Scale.Quality of Life Research, 22, 2581-2594.

- Varni, J.W., Burwinkle, T.M., & Szer, I.S. (2004). The PedsQL™ Multidimensional Fatigue Scale in pediatric rheumatology: Reliability and validity. Journal of Rheumatology; 31, 2494-2500.

- Varni, J.W., & Limbers, C.A. (2008). The PedsQL™ Multidimensional Fatigue Scale in young adults: Feasibility, reliability and validity in a university student population. Quality of Life Research, 17, 105-114.

- Panepinto, J.A., Torres, S., Bendo, C.B., McCavit, T.L., Dinu, B., Sherman-Bien, S., Bemrich-Stolz, C., & Varni, J.W. (2014). PedsQL™ Multidimensional Fatigue Scale in sickle cell disease: Feasibility, reliability and validity.Pediatric Blood & Cancer, 61, 171–177.

Neurofibromatosis Type 1 Module:

- Nutakki, K., Hingtgen, C.M., Monahan, P., Varni, J.W., & Swigonski, N.L. (2013). Development of the adult PedsQL™ Neurofibromatosis Type 1 Module: Initial feasibility, reliability and validity.Health and Quality of Life Outcomes, 11:21, 1-9

Neuromuscular Module:

- Iannaccone, S.T., Hynan, L.S., Morton, A., Buchanan, R., Limbers, C.A., & Varni, J.W. (2009). The PedsQL™ in pediatric patients with Spinal Muscular Atrophy: Feasibility, reliability, and validity of the Pediatric Quality of Life Inventory™ Generic Core Scales and Neuromuscular Module.Neuromuscular Disorders, 19, 805-812.

- Davis, S.E., Hynan, L.S., Limbers, C.A., Andersen, C.M., Greene, M.C., Varni, J.W., & Iannaccone, S.T. (2010). The PedsQL™ in pediatric patients with Duchenne Muscular Dystrophy: Feasibility, reliability, and validity of the Pediatric Quality of Life Inventory™ Neuromuscular Module and Generic Core Scales.Journal of Clinical Neuromuscular Disease, 11, 97-109.

Oral Health Scale:

- Steele, M.M., Steele, R.G., & Varni, J.W. (2009). Reliability and validity of the PedsQL™ Oral Health Scale: Measuring the relationship between child oral health and health-related quality of life.Children’s Health Care, 38, 228-224.

Pediatric Pain Coping Inventory™:

- Varni, J.W., Waldron, S.A., Gragg, R.A., Rapoff, M.A., Bernstein, B.H., Lindsley, C.B., & Newcomb, M.D



© Mapi Research Trust. The unauthorized modification and use of any portion of this document is prohibited.

(1996). Development of the Waldron/Varni Pediatric Pain Coping Inventory. Pain, 67, 141-150.

Pediatric Pain Questionnaire:

- Varni, J.W., Thompson, K.L., & Hanson, V. (1987). The Varni/Thompson Pediatric Pain Questionnaire: I. Chronic musculoskeletal pain in juvenile rheumatoid arthritis.Pain, 28, 27-38.

Present Functioning Visual Analogue Scales:

- Sherman, S.A., Eisen, S., Burwinkle, T.M., & Varni, J.W. (2006). The PedsQL™ Present Functioning Visual Analogue Scales: Preliminary reliability and validity.Health and Quality of Life Outcomes, 4:75, 1-10.

Sickle Cell Disease Module:

- Panepinto, J.A., Torres, S., Bendo, C.B., McCavit, T.L., Dinu, B., Sherman-Bien, S., Bemrich-Stolz, C., & Varni, J.W. (2013). PedsQL™ Sickle Cell Disease Module: Feasibility, reliability and validity.Pediatric Blood & Cancer, 60, 1338–1344.

- Panepinto, J.A., Torres, S., & Varni. J.W. (2012). Development of the PedsQL™ Sickle Cell Disease Module items: Qualitative methods.Quality of Life Research, 21, 341-357.

Stem Cell Transplant Module:

- Lawitschka, A., Güclü, E.D., Varni, J.W., Putz, M., Wolff, D., Pavletic, S., Greinix, H., Peters, C., & Felder-Puig, R. (2014). Health-related quality of life in pediatric patients after allogeneic SCT: Development of the PedsQL™ Stem Cell Transplant Module and results of a pilot study. Bone Marrow Transplantation, 49, 1093–1097.

Rheumatology Module:

- Varni, J.W., Seid, M., Knight, T.S., Burwinkle, T.M., Brown, J., & Szer, I.S. (2002). The PedsQL™ in pediatric rheumatology: Reliability, validity, and responsiveness of the Pediatric Quality of Life Inventory™ Generic Core Scales and Rheumatology Module.Arthritis and Rheumatism, 2002; 46: 714-725.

Transplant Module:

- Weissberg-Benchell, J., Zielinski, T.E., Rodgers, S., Greenley, R.N., Askenazi, D., Goldstein, S.L., Fredericks, E.M., McDiarmid, S., Williams, L., Limbers, C.A., Tuzinkiewicz, K., Lerret, S., Alonso, E.M., & Varni, J.W. (2010).



© Mapi Research Trust. The unauthorized modification and use of any portion of this document is prohibited.

Pediatric health-related quality of life: Feasibility, reliability and validity of the PedsQL™ Transplant Module.American Journal of Transplantation, 10, 1677-1685.



© Mapi Research Trust. The unauthorized modification and use of any portion of this document is prohibited.
