# Supplementary material for: Morbidity and Complications of Diabetes Mellitus in Children and Adolescents in Ghana: Protocol for a Longitudinal Study
Source: JMIR Res Protoc. 2021 Jan 6;10(1):e21440. doi: 10.2196/21440 (PMC7817364; doi:10.2196/21440)
Supplement: Multimedia Appendix 5 [file resprot_v10i1e21440_app5.pdf]

SCHOOL OF MEDICINE AND DENTISTRY
COLLEGE OF HEALTH SCIENCES
UNIVERSITY OF GHANA
ACADEMIC AFFAIRS OFFICE

Phone: +233-0302-666987-8
Fax: +233-0302-663062

E-mail: academic.ugms@chs.edu.gh

P O Box 4236
Accra
Ghana

My Ref. No: MS-AA/C.2/Vol.18^A^

25th August, 2014

Your Ref. No.

Dr. Vera Adobea Essuman
Department of Surgery, Eye Unit
School of Medicine and Dentistry
Korle-Bu

ETHICAL CLEARANCE

Protocol Identification Number: MS-Et/M.12 – P 4.5/2013-2014

The Ethical and Protocol Review Committee of the University of Ghana Medical School on 22nd August, 2014 unanimously approved your research proposal.

TITLE OF PROTOCOL:"**Morbidity Complication for Diabetes Mellitus in Ghanaian Children and Adolescents**

PRINCIPAL INVESTIGATOR: **Dr. Vera Adobea Essuman**

This approval requires that you submit six-monthly review reports of the protocol to the Committee and a final full review to the Ethical and Protocol Review Committee at the completion of the study. The Committee may observe, or cause to be observed, procedures and records of the study during and after implementation.

Please note that any significant modification of this project must be submitted to the Committee for review and approval before its implementation.

You are required to report all serious adverse events related to this study to the Ethical and Protocol Review Committee within seven (7) days verbally and fourteen (14) days in writing.

As part of the review process, it is the Committee's duty to review the ethical aspects of any manuscript that may be produced from this study. You will therefore be required to furnish the Committee with any manuscript for publication.

This ethical clearance is valid till 30th September, 2015.

Further renewal of approval will be given upon presentation of an annual report of work done.

Please always quote the protocol identification number in all future correspondence in relation to this protocol.

Signed: Welbeck ....
PROFESSOR JENNIFER WELBECK
(CHAIRPERSON, ETHICAL AND PROTOCOL REVIEW COMMITTEE)

cc: Ag. Dean
Head of Department
[illegible]
